# Differentiated transcriptional signatures in the maize landraces of Chiapas, Mexico

**DOI:** 10.1186/s12864-017-4005-y

**Published:** 2017-09-08

**Authors:** Matthew A. Kost, Hugo R. Perales, Saranga Wijeratne, Asela J. Wijeratne, Eric Stockinger, Kristin L. Mercer

**Affiliations:** 1 Department of Horticulture and Crop Science, The Ohio State University/Ohio Agricultural Research and Development Center (OARDC), Wooster, OH USA; 2 El Colegio de la Frontera Sur, Departmento de Agroecología, San Cristóbal de Las Casas, Chiapas Mexico; 3 Molecular Cellular and Imagining Center, The Ohio State University/OARDC, Wooster, OH USA; 4 Department of Horticulture and Crop Sciences, The Ohio State University, Columbus, OH USA; 5 Department of Biological Sciences, Arkansas State University, Jonesboro, AR USA

**Keywords:** Maize, Landraces, Transcriptomics, WGCNA, RNA-seq, Chiapas, Adaptation

## Abstract

**Background:**

Landrace farmers are the keepers of crops locally adapted to the environments where they are cultivated. Patterns of diversity across the genome can provide signals of past evolution in the face of abiotic and biotic change. Understanding this rich genetic resource is imperative especially since diversity can provide agricultural security as climate continues to shift.

**Results:**

Here we employ RNA sequencing (RNA-seq) to understand the role that conditions that vary across a landscape may have played in shaping genetic diversity in the maize landraces of Chiapas, Mexico. We collected landraces from three distinct elevational zones and planted them in a midland common garden. Early season leaf tissue was collected for RNA-seq and we performed weighted gene co-expression network analysis (WGCNA). We then used association analysis between landrace co-expression module expression values and environmental parameters of landrace origin to elucidate genes and gene networks potentially shaped by environmental factors along our study gradient. Elevation of landrace origin affected the transcriptome profiles. Two co-expression modules were highly correlated with temperature parameters of landrace origin and queries into their ‘hub’ genes suggested that temperature may have led to differentiation among landraces in hormone biosynthesis/signaling and abiotic and biotic stress responses. We identified several ‘hub’ transcription factors and kinases as candidates for the regulation of these responses.

**Conclusions:**

These findings indicate that natural selection may influence the transcriptomes of crop landraces along an elevational gradient in a major diversity center, and provide a foundation for exploring the genetic basis of local adaptation. While we cannot rule out the role of neutral evolutionary forces in the patterns we have identified, combining whole transcriptome sequencing technologies, established bioinformatics techniques, and common garden experimentation can powerfully elucidate structure of adaptive diversity across a varied landscape. Ultimately, gaining such understanding can facilitate the conservation and strategic utilization of crop genetic diversity in a time of climate change.

**Electronic supplementary material:**

The online version of this article (doi:10.1186/s12864-017-4005-y) contains supplementary material, which is available to authorized users.

## Background

Maize was domesticated from *Zea mays* ssp. *parviglumis* in the Balsas River Valley of Mexico 9000 to 10,000 years ago [1–3]. There are around 59 phenotypic classes, or races, of maize in Mexico [4] and maize landraces (i.e., traditional varieties) of Mexico contain the highest number of alleles per locus of any country in the Americas [5]. General factors contribute to the high level of maize diversity in Mexico, including: (i) being the center of crop origin [6]; (ii) gene flow between wild teosinte species and maize landrace populations [7–9]; (iii) diverse selection previously imposed by ecological and environmental heterogeneity [10, 11]; and (iv) the cultural diversity present among Mexican maize landrace farmers [7, 11, 12]. These factors, along with neutral evolutionary processes, have shaped the morphological, phenological, and physiological characteristics of maize throughout Mexico [13], as well as the genetic diversity underlying these traits.

Farmers in the southernmost state of Chiapas, Mexico, and the surrounding region, grow a diversity of maize. They maintain upwards of 20 races of maize, 11 of which are common [14], and three are dominant [15]. The distribution of maize races in southern Mexico and Guatemala is strongly determined by environmental factors, which vary with elevation [15, 16]*.* As is the case for many crops, culture also influences the distribution and differentiation of maize landraces in the region [15–21]. However, phenotypic differentiation has not always corresponded with genetic differentiation in these studies, a fact that warrants study of ‘selected variation’, or differentiation induced by natural selection [17, 18]. Mercer et al. [22] provided evidence that elevation has shaped maize landrace diversity in Chiapas producing patterns of local adaptation. This has provided a foundation for determining the underlying mechanisms of local adaptation in the maize landraces along the study gradient, including developmental timing (Mercer et al. under review) and physiology (Pace et al. unpublished manuscript).

Differential global gene expression analysis performed on differentially adapted populations, grown together in a common garden, provides a way to identify genes putatively involved in adaptation. In some cases, the planting of diverse populations in common gardens can illuminate genetic differences underlying phenotypes, such as gene expression. Such an approach with diverse populations of Sitka spruce [23] identified genes putatively relevant to cold acclimation. Differentially expressed (DE) genes that were overrepresented in gene ontology (GO) abiotic stress categories or that had functional annotations for cold acclimation provided fodder for further validation. Similarly, in maize, Hayano-Kanashiro et al. [24] performed a microarray study using three Mexican landraces originating from different moisture environments to identify putative candidate genes underlying drought tolerance. RNA-seq can now be employed to investigate DE genes [25–28]; however a reductionist approach of investigating DE genes individually may not be ideal for gaining insight into quantitative traits, such as those involved in adaptation to abiotic and biotic stresses.

By contrast, weighted gene co-expression network analysis (WGCNA), a powerful bioinformatics tool, may more holistically determine how the environment shapes genetic diversity across the landscape. Co-expression network analyses provide a systems biology perspective on the transcriptomes of biological samples by identifying gene modules co-expressed among samples [29]. Genes within a co-expressed module are assumed to be co-regulated and involved in the same biological function or pathway, a phenomenon of ‘guilt by association’ [30]. A number of studies have used WGCNA to identify correlations between expression values representative of each module and variation in trait values across biological samples [31–34]. This type of analysis can lead to the identification of modules underlying study traits and can be followed up with GO enrichment analysis and/or the identification of ‘hub’ genes (i.e., highly connected genes in pathways/networks). To identify how the environment shapes genetic diversity across the landscape, we propose that environmental parameters of population origin can be used as the “traits” in module eigengene – trait analyses. Such analyses may elucidate: (i) environmental factors that have led to differentiation among samples; and (ii) potential adaptations to particular environmental conditions, whether governed by single genes or highly connected ‘hub’ genes that can influence entire gene networks.

We conducted a landscape level RNA-seq analysis on maize landraces collected along an elevational gradient in Chiapas, Mexico, where Mercer et al. [22] reported signals of local adaptation. A total of 15 landraces, five from each of three elevational zones (highland, 2100 m; midland, 1550 m; lowland, 600 m), were planted in a midland common garden at 1531 m. RNA-seq libraries were generated using leaf tissue collected early in the growing season. Differential expression analysis, principal component analysis (PCA) and WGCNA were then conducted to address the following objectives. (i) Determine if elevation of landrace origin shaped the gene expression profiles of the 15 maize landraces. (ii) Identify correlations between co-expression modules and environmental parameters of landrace origin. (iii) Identify enriched GO categories and functions of ‘hub’ genes in modules exhibiting strong correlations with environmental parameters of landrace origin. As a whole, these analyses aimed to elucidate how environmental differences along the elevational gradient may have structured genetic diversity involved in local adaptation.

## Methods

### Study region and maize landrace collections

Chiapas, the southernmost state of Mexico, constitutes a prime location to study how natural selection has shaped genetic diversity in maize landraces. From the highlands of the Sierra Madre de Chiapas to the lowlands of the Mexico-Guatemala border, near Frontera Comalapa, exists an elevational gradient of more than 2000 m. Accompanying this elevational gradient are both abiotic and biotic gradients [35]. Temperature tends to negatively correlate with elevation and is known to organize maize diversity at larger scales [10].

Chiapas maize landrace seeds employed in this study were provided by farmers along this same elevational gradient—collections were performed in 2009. Communities sampled fell into one of three distinct elevational zones: highland (~ 2100 m; ranging from 2060 to 2153 m); midland (~ 1550 m; ranging from 1531 to 1584 m); and lowland (~ 600 m; ranging from 563 to 684 m). Five landraces were collected from each of the three zones for a total of 15 landraces (Table 1). When collecting a landrace population from a given farmer we requested at least 100 maize ears to ensure a representative sample. The 15 landraces belonged to four maize races. The highland landraces were either *Olotillo *or *Olotón*; midland landraces were all *Comiteco*; and all lowland landraces consisted of *Tuxpeño *(Table 1).

**Table 1:** Chiapas, Mexico maize landraces provided by landrace farmers

Elevation	Landrace ID	Municipality/Community	Latitude, longitude	Elevation (m)	Race
Lowland	1	Chicomuselo/Raizal	15.8939 N, 92.2537 W	648	Tuxpeño
	4	Frontera Comalapa/Benito Juárez	15.8229 N, 92.2042 W	563	Tuxpeño
	6	Frontera Comalapa/Benito Juárez	15.8229 N, 92.2042 W	563	Tuxpeño
	7	La Trinitaria/Juan Aldama	15.8554 N, 91.9377 W	598	Tuxpeño
	9	La Trinitaria/Nuevo Llano Grande	15.8390 N, 91.9363 W	595	Tuxpeño
Midland	10	La Trinitaria/El Rosario Tierra Blanca	16.0770 N, 91.7461 W	1533	Comiteco
	12	La Trinitaria/Miguel Hidalgo	16.1056 N, 91.7780 W	1524	Comiteco
	13	Comitán de Domínguez/San Francicsco El Ricón	16.2814 N, 92.1357 W	1584	Comiteco
	17	Las Margaritas/Ignacio Zaragoza	16.3515 N, 91.9194 W	1531	Comiteco
	18	Las Margaritas/Ignacio Zaragoza	16.3515 N, 91.9194 W	1531	Comiteco
Highland	20	Comitán de Domínguez/Ignacio Zaragoza	16.3612 N, 92.1792 W	2089	Olotillo
	26	San Cristobal de Las Casas/Carrizal	16.6714 N, 92.6544 W	2153	Olotón
	27	San Cristobal de Las Casas/Carrizal	16.6735 N, 92.6618 W	2137	Olotón
	29	Teopisca/San Isidro Chichiuistán	16.5979 N, 92.5656 W	2060	Olotón
	30	Teopisca/San Isidro Chichiuistán	16.6021 N, 92.5591 W	2060	Olotón

Elevation, elevational zone collected from; Landrace ID, arbitrary ID given to each landraces; Municipality/Community, locations where landraces were collected; Latitude, longitude, precise location of collections; Elevation (m), elevation in meters of where collections were obtained; Race, the race category of the landrace

### Environmental parameters

For the WGCNA module – environmental parameter analysis we used thirty years of data (1971–2000) on seven temperature-, three precipitation-, and one evaporation-related environmental parameters, collected from weather stations nearest each of the landrace collection sites (Comisión Nacional del Agua – Servicio Meteorológico Nacional, México; www.smn.cna.gob.mx/es/). For detailed information on weather station locations see Additional file 1. Annual averages for each parameter were calculated and used in subsequent analyses. Definitions of all environmental parameters are in Additional file 2. Although we used all eleven environmental parameters in our analyses, we are more confident about results related to the temperature parameters. This is because: (i) the accuracy of the precipitation- and evaporation- related parameters may be less due to collection methods; and (ii) had our focus been on precipitation and evaporation we would likely have selected a study area with greater gradients for these parameters.

### Common garden design and tissue collection

In 2011, we planted a common garden in the midland elevational zone (1531 m) to provide a uniform environment in which to assay gene expression differences and correlate them with environmental conditions at the origin of each landrace. The midland garden in 2011 provided beneficial growing conditions where survival was uniformly high for all types. While common garden experiments do allow researchers to isolate genetic differences among groups of plants, they can be differentially benign to plants of different origins, affecting phenotypes, including seed production or gene expression. Only reciprocal transplant experiments, which were outside the scope of this study, can remedy that by discerning genetic variation at local and non-local sites for each type and any genotype by environment interactions influencing the phenotype of interest.

All 15 maize landraces were sown in a modified split-plot design at the midland garden. Randomized main plots were assigned a given elevation of origin for landraces, and individual landraces were randomly assigned to subplots within the appropriate main plot. This modification was necessary due to the difference in growth and phenology of landraces from the different elevations. Each landrace was sown in a row made up of 10 *matas* (planting locations) containing three seeds, totaling 30 seeds planted per landrace per block. Each main plot was surrounded by an edge row of landrace seeds from that main plot’s elevation to reduce edge effects. There were three blocks.

We collected the most newly emerged leaf possessing a leaf collar early in the growing season (24 June 2011, ~ 3rd leaf stage) on three randomly selected plants per landrace per block for a total of nine individuals sampled per landrace and 135 plants overall. We immediately froze tissue in liquid nitrogen to conserve ribonucleic acid (RNA) integrity and stored the samples at − 80 °C until we performed RNA extractions. Leaf tissue was chosen since responses to temperature, precipitation, and evaporation (as well as ultraviolet B (UVB), analyzed elsewhere) are often mediated through the leaf.

### RNA extraction, library preparation and sequencing

RNA extractions and RNA-seq library construction were performed on all collected leaf tissue. To gain a representative landrace-level RNA expression profile, we pooled RNA from the three individuals collected per landrace per block before library preparation. The 45 RNA-seq libraries maintained the field replication structure. RNA-seq libraries were constructed as in Zhong et al. [36]. The 45 RNA-seq libraries were sequenced in four flow cell lanes of the Illumina HiSeq2500 (12 libraries per lane with paired end sequencing at 50 base pairs (bp)).

### Library quality, read trimming, and mapping

A version of Galaxy [37] maintained by the Molecular and Cellular Imaging Center Computation Biology Laboratory (MCBL) at the Ohio Agricultural Research and Development Center (OARDC) facilitated the implementation of software to determine library quality and to perform read trimming. All settings were maintained as default with exceptions noted. *FastQC* version 0.10.1 [38] was used for general statistics and for read quality and quantity determination before and after cleaning of RNA-seq reads. *Cutadapt* version 0.9.5.a [39] was used to remove polyA/polyT tails and adapter sequences. Quality trimming was executed in Galaxy [40] with the *Trim the reads by quality* (version 1.2.2). The minimum length threshold was set to 25 because of our short, 50 bp read size.

Preprocessed libraries were mapped to the maize B73 v2 5b.60 genome, which was downloaded from Phytozome (www.phytozome.net) [41], with the splice junction mapper *Tophat2* version 2.0.10 [42]. *Tophat2* settings were kept at default with the following exceptions: (i) mean inner distance between mate pairs and standard deviation (s.d.) for distance between mate pairs set to 150 bp; (ii) maximum and minimum intron length set to 20,000 bp and 70 bp, respectively; and (iii) maximum and minimum intron length that may be found during split-segment search set to 20,000 bp and 50 bp, respectively. The final setting for read mismatches was maintained at two per read. Since differential mapping may occur among RNA-seq libraries due to genetic differentiation among the populations used to create them when mapping to the same reference genome, we tested for this by performing a second mapping run with read mismatches set to zero. We then used the Zmays_191_gene.gff3 (www.Phytozome.net) annotation file and *htseq-count* version 0.6.1 [43] to determine the number of reads mapping to each gene. Raw read counts from each of the 45 RNA-seq libraries were then compiled into a counts matrix for subsequent analysis.

### Differential expression and VST counts

*DESeq2* version 2.13 [44] was used for both differential expression analysis between highland and lowland maize landraces and for generation of variance stabilized transformed (VST) counts for downstream analysis. We imported raw read counts for all 45 libraries into *DESeq2*. Our differential expression analysis model was constructed with both elevation of landrace origin and block (Design = ~Block + Landrace origin) so that variance due to block could be accounted for as we focused on differential expression due to elevation of landrace origin. Genes exhibiting an adjusted false discovery rate (FDR) of 0.05 or less were considered DE. We also used *DESeq2* to generate homoscedastic VST counts for all libraries, which were subsequently used for both PCA and downstream WGCNA*.* We then used *DESeq2* to generate a PCA plot of all our libraries. VST counts for each landrace were averaged across block in preparation for WGCNA.

### Weighted correlation network analysis (WGCNA)

We used the weighted correlation network analysis (*WGCNA*) R package [45], to identify: (i) gene co-expression modules across our 15 maize landraces; and (ii) module eigengene value – environmental associations. VST data for all annotated genes in our 15 libraries were loaded into *WGCNA*. The similarity matrix was raised to the power of seven to identify co-expression modules. Once modules were formed, we sought to identify those having strong correlations with environmental parameters of landrace origin; these environmental parameters were inputted into our *WGCNA* data frame as landrace “traits”. For each of the 44 identified co-expression modules (Additional file 3), eigengene values were generated for each of the 15 landraces. Pearson correlations and their associated *p*-values were then generated for all pairwise comparisons of the 44 module eigengene expression values across the 15 landraces and the 11 environmental parameter values of landrace origin. Bonferroni adjustments corrected for multiple comparisons (*n* = 484). We were unable to account for genetic structure in our analysis using RNA-seq data due to the pooling of our samples and our small sample size (i.e., for each landrace we sequenced three pooled samples where RNA from three individual plants was bulked = 9 samples). Several modules exhibiting high correlations with temperature parameters were further studied to identify: (i) module expression patterns across the elevations where landraces originated; (ii) overrepresented GO categories; and (iii) highly connected module ‘hub’ genes related to temperature parameters of landrace origin. Thus, we focus here on two co-expression modules highly correlated with temperature related variables (turquoise & yellow).

The correlations between the turquoise module and maximum normal mean temperature and the yellow module and minimum daily mean temperature were the greatest and most significant correlations between modules and temperature related environmental variables (see results below). In order to determine the biological functions of these environmentally relevant modules, we extracted turquoise module genes along with their module membership (MM) and gene significance (GS) values for maximum normal mean temperature; we also extracted yellow module genes, MM values, and GS values for minimum daily mean temperature. GS values are the correlations between the expression value of single genes and environmental trait values across samples. By contrast, MM values are the correlations between single gene expression values and module eigengene values across samples. Genes from each module were screened for enriched GO categories using *AGRIGO* and were also used for ‘hub’ gene analyses (see below).

Genes belonging to the ≥85th percentile for both MM and GS for both module – temperature parameter correlations were retained for subsequent inquiry as these genes are likely ‘key drivers’ (i.e. ‘hub’ genes) within the pathways making up co-expression modules [46]. 85% has been used in WGCNA analyses e.g., [46] as a useful cut-off for focusing on the most highly connected genes. 85th percentile genes from both modules were functionally annotated using Phytozome [41] and MaizeGDB [47]. We obtained gene name information from The Arabidopsis Information Resource (TAIR) [48]. TAIR descriptions were used to assign ‘hub’ genes to ‘Functional Category’ (see Table 2 & Additional file 4). For ‘hub’ genes lacking descriptions we searched the literature for their putative functions. Finally, maize transcription factor information was collected from GrassTFDB on the Grass Regulatory Information Server GRASSIUS [49, 50].

## Results

### Read counts and mapped reads

We assessed read quality and quantity for each of the 45 RNA-seq libraries in preparation for mapping to the maize reference genome. Using *FastQC*, we confirmed libraries were of high quality both before and after reads were preprocessed. Raw read counts ranged from approximately five to 21 million per library (majority nine to 14 million; Additional file 4). After trimming, paired-end reads ranged from five to 20 million, showing that a majority of reads were still paired.

By independently mapping the preprocessed reads from each library to the maize reference genome, we found that between 70 and 80% of the initial raw reads from each library uniquely mapped to the maize genome when allowing a two base pair mismatch (except for lowland landrace 6 (block 2) with 66%; calculated from Additional file 5; uniquely mapped reads/raw reads). When allowing zero mismatches, the mapping percentages declined to between 61 to 72% and were not significantly different (α = 0.05; data not shown), suggesting that elevation of origin did not influence mapping percentages. During the remainder of our analyses we used results from the two base pair mismatch mapping run—the default parameter value in *TopHat2*.

### Elevation has shaped the maize landrace transcriptomes

**Fig. 1:**
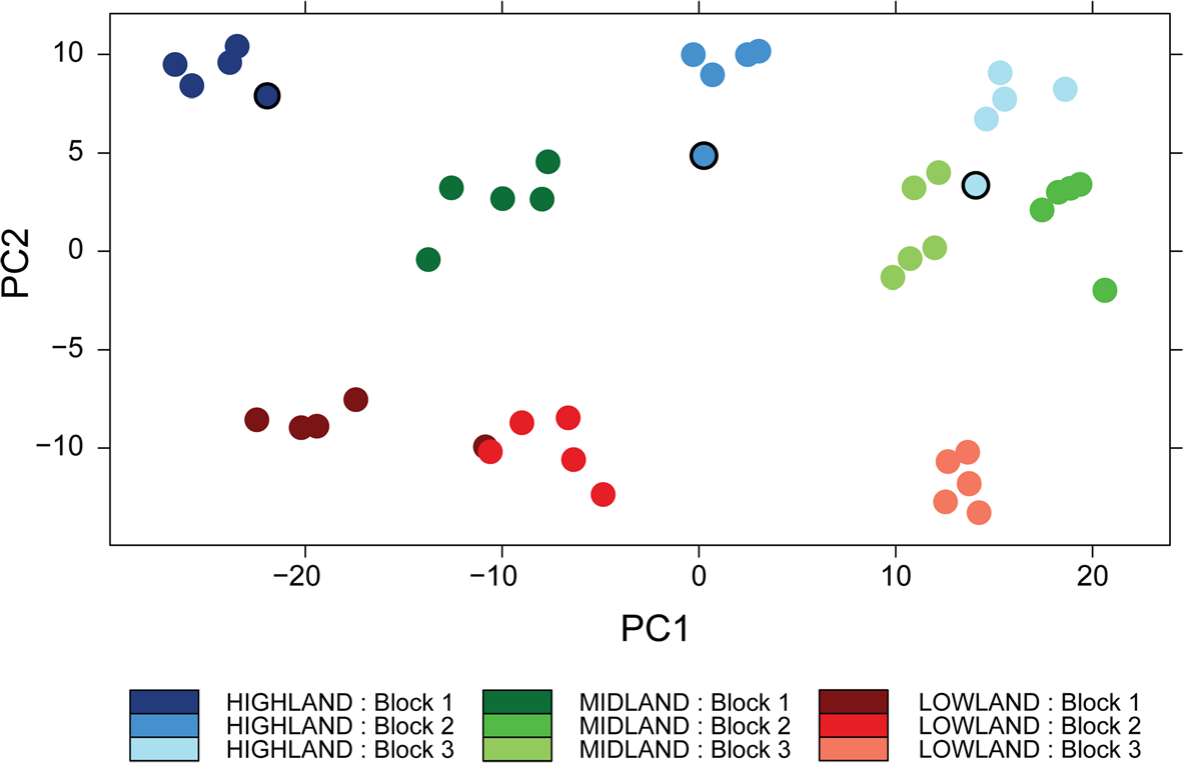
PCA plot of the 45 pooled maize RNA-seq libraries. Blocks (replicates) of a given elevation are represented by different hues of a color. Highland landraces outlined in black are replicates from highland landrace 20 (*Olotillo*)

To test whether elevation of landrace origin was associated with differences in transcript profiles we carried out PCA. The first principal component (PC1) identified in our PCA grouped the 45 RNA-seq libraries by replication (i.e., spatial block) while the second (PC2) grouped them by elevation of landrace origin (Fig. 1). While all elevations of landrace origin were clearly distinguished, highland and midland maize landraces grouped with each other to a greater extent than either did with the lowland landraces. Highland landrace 20, the only *Olotillo *(Table 1), was more similar in its expression profile to the midland landraces than were the remaining four highland landraces. These findings suggest that elevation of landrace origin has influenced the transcriptome profiles of the maize landraces.

### Environmental variation

**Fig. 2:**
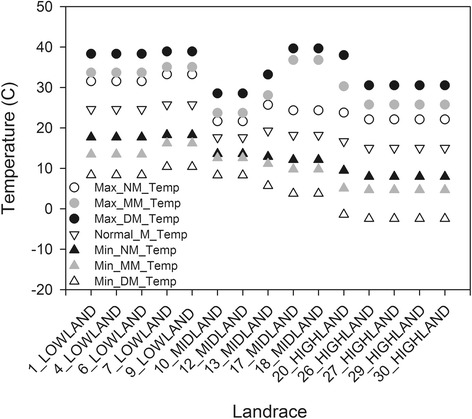
Thirty year averages of temperature -related environmental parameters of maize landrace origin. Landrace number and elevational zone are on the x-axis and environmental parameters are along the y-axis. Abbreviations: C – Celsius, DM – daily mean, M – mean, MM – monthly mean, NM – normal mean. See Additional file 2 for definitions for each environmental parameter abbreviation

To determine whether environmental factors of landrace origin other than elevation varied along our study gradient we queried 11 climatic parameters in the region (Additional file 2). We observed a salient pattern of a decrease in temperature, precipitation, and evaporation with an increase in elevation of landrace origin (Fig. 2 & Additional file 6). Despite this broad pattern, other forms of environmental variation can be seen across the study area likely due to particular landscape features (Additional file 7).

### 44 co-expression modules identified

Co-expression modules are groups of highly interconnected nodes (i.e., genes) exhibiting similar gene expression patterns [51]. Using WGCNA, we found 44 co-expression modules among the 15 maize landraces collected from the three elevational zones in Chiapas, Mexico (Additional file 3). The co-expression modules contained between 44 and 4794 genes and satisfied approximate scale-free topology (Additional file 8). Importantly, co-expression modules may be expressed at different intensities across samples. Thus, the modules that we identified were co-expressed in all 15 maize landraces, albeit, as discussed below, at different levels in different landraces. The genes within a given module were assigned a common ‘module color’ (Additional file 3).

### Environmental parameter – Module correlations

**Fig. 3:**
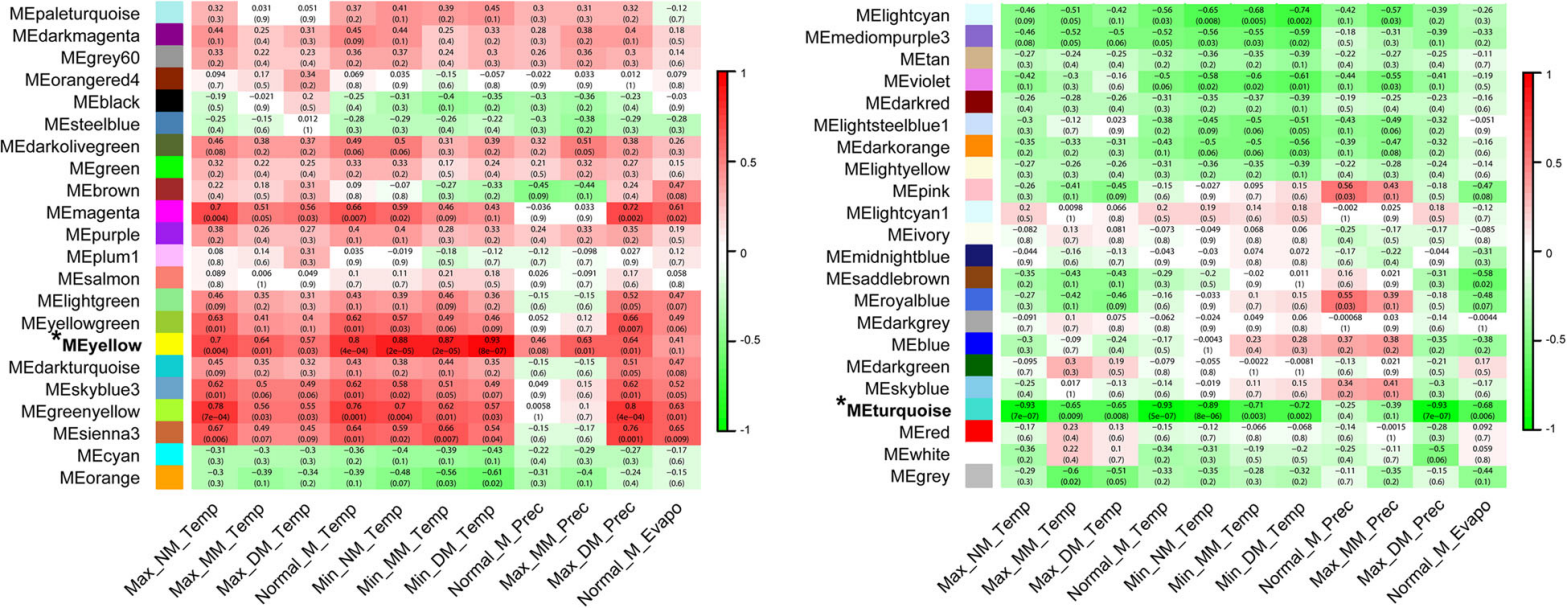
Heat map of environmental parameter – module eigengene correlations. Pearson correlation coefficients and *p*-values (in parentheses) are reported. Intensity and direction of correlations are indicated on the right side of the heat map (*red*, positively correlated; *green*, negatively correlated). The turquoise and yellow modules are marked with an * and are in bold. A *p*-value of 0.05, when adjusted for multiple comparisons using the Bonferroni method (*n* = 484) is equivalent to a value of 1.03e-4 here

To identify co-expression modules exhibiting strong correlations with environmental parameters of landrace origin, we searched for significant correlations among these parameters and module eigengene expression values. We observed many strong correlations; 16 of the 44 modules correlated with at least one environmental parameter at a raw *p* < 0.05 significance level, three had parameters with correlations significant at a raw *p* < 0.001 level (Fig. 3). The turquoise and yellow co-expression modules had the greatest number and strength of correlations to environmental traits. In fact, they were the only two modules retaining significant correlations with any of the environmental parameters after Bonferroni adjustment for multiple comparisons (*p* < 1.03e-4 in Fig. 3 are considered significant at <0.05 after Bonferroni adjustment). For the yellow module, only correlations with the three minimum temperature parameters (for all three, *r* > 0.87) remained significant after adjusting the *p*-value cut-off (Fig. 3). The most significant correlation for the yellow module was with minimum daily mean temperature (Fig. 3; *r* = 0.93, *p* = 8e-07, Bonferroni corrected *p*-value = 0.00039). For the turquoise module, three of the four environmental parameters most negatively correlated (*r* < −0.89) were temperature related (maximum normal mean temperature, normal mean temperature and minimum normal mean temperature), and the fourth was maximum daily mean precipitation. The greatest and most significant correlations in the turquoise module were with maximum normal mean temperature and maximum daily mean precipitation (Fig. 3; both *r* = −0.93, *p* = 7e-07, Bonferroni corrected *p*-value = 0.00034). Due to the strength of the correlations between these environmental parameters and the turquoise and yellow modules, we focus our attention here. Since each of these modules had some of their strongest correlations with temperature related parameters, and the fact that we were more confident with the temperature related variables, we limit our subsequent analyses to investigating their correlations with maximum normal mean temperature (turquoise) and minimum daily mean temperature (yellow). However, it is important to note that for the turquoise module, maximum daily mean precipitation, an indicator of extreme precipitation events, may also be worthy of study due to its high correlation with the module.

### Turquoise/yellow module expression among landraces and GO enrichment

**Fig. 4:**
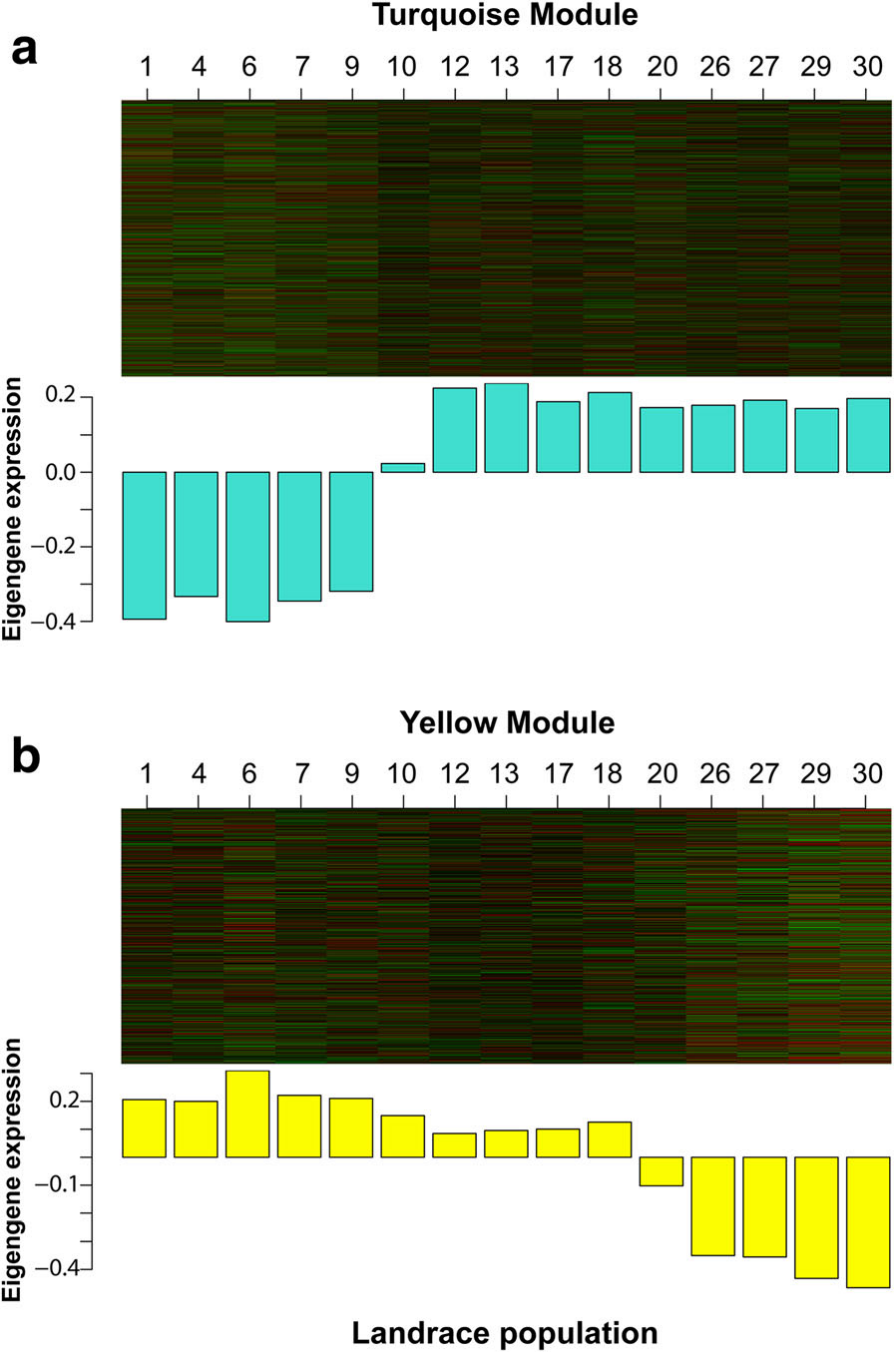
Turquoise (**a**) and yellow (**b**) module eigengene expression values across the 15 maize landraces. The upper portions of both figures are heat maps of the expression values of each gene (row) in each maize landrace population (column): *red* – highly expressed; *green* – lowly expressed; *black* – neutral

Next, we sought to clarify the extent that elevation of landrace origin influenced module expression values of the environmentally relevant turquoise and yellow modules. Since module eigengene expression values are the first principal components of modules [45], they provide insight into the behavior of a given module across a set of biological samples. If a majority of the module genes of a given sample (i.e., here, a given maize landrace) are under-expressed, then the eigengene value will be negative (i.e., down-regulated). Alternatively, if they are over-expressed, then it will be positive (i.e., up-regulated). All five lowland maize landraces exhibited negative eigengene values for the turquoise module, while the midland and highland landraces had positive eigengene values (Fig. 4a). The yellow module eigengene values for the lowland and midland landraces were all positive with lowlands generally having higher values than midland landraces. The highland landraces on the other hand all exhibited negative eigengene values for the yellow module (Fig. 4b). These results are consistent with the finding of the turquoise and yellow modules being correlated with clinal environmental variation (e.g., as found with temperature).

We performed GO enrichment analysis on genes making up the turquoise and yellow modules to elucidate general biological processes in which these modules were involved. Both the turquoise and yellow modules were enriched for GO terms. The 4470 genes within the turquoise module were enriched for four GO terms: cellular nitrogen compound metabolic process, catalytic activity, coated membrane and membrane coat (Additional file 9). The 1862 genes present in the yellow module were enriched for 15 GO terms, five of which were descendent (i.e. detailed) GO terms. The descendent GO terms included adenosine triphosphate (ATP) binding, protein tyrosine kinase activity, calcium ion binding, protein serine/threonine kinase activity and oxidoreductase activity (Additional file 9).

### Identification and functional annotation of ‘hub’ genes

Since ‘hub’ genes are centrally located within coexpression modules (i.e., highly connected) that often represent biological pathways and/or networks [45], they can provide insight into the biological function(s) of modules and can lead to the identification of transcription factors involved in the regulation of these functions [34]. As the turquoise and yellow modules were the most relevant to temperature (Fig. 3), we queried these modules for ‘hub’ genes relevant to maximum normal mean temperature and minimum daily mean temperature of landrace origin, respectively.

**Table 2:** ‘Hub’ genes of interest. Turquoise module ‘hub’ genes upregulated in the highland (A) and lowland (B) landraces; yellow module ‘hub’ genes upregulated in the highland (C) and lowland (D) landraces organized by functional categories

Functional category	Maize gene ID	MaizeTF	TAIR symbol	Arabidopsis TAIR10 definition
A. Turquoise module – Highland landraces				
Transcription	GRMZM2G010920	ZmGLK18	-	myb-like HTH transcr. Regulator family
	GRMZM2G097275	ZmSBP27	SPL2	squamosa promoter binding protein-like 2
	GRMZM2G154169	-	GIF3	GRF1-interacting factor 3
	GRMZM2G398124	-	TAF9	TATA binding protein associated factor 21 kDa subunit
	GRMZM2G414141	-	-	transcription coactivators
	GRMZM5G828396	ZmbHLH81	CIB2	basic helix-loop-helix (bHLH) DNA-binding superfamily protein
Kinases	GRMZM2G053117	-	MPK20	MAP kinase 20
	GRMZM2G079303	-	RLP48	receptor like protein 48
	GRMZM2G144028	-	LRK10L1.2	Protein kinase superfamily protein
	GRMZM2G144245	-	NARA5	pfkB-like carbohydrate kinase family protein
	GRMZM2G160853	-	-	S-locus lectin protein kinase family protein
	GRMZM2G473104	-	NARA5	pfkB-like carbohydrate kinase family protein
	GRMZM2G049510	-	KIP1	Kinase interacting (KIP1-like) family protein
B. Turquoise module – Lowland landraces				
Transcription	GRMZM2G033413	ZmbZIP100	ABF4	ABRE binding factor 4
	GRMZM2G035103	-	ZAT10	salt tolerance zinc finger
	GRMZM2G067624	ZmSBP29	SPL4	squamosa promoter binding protein-like 4
	GRMZM2G079727	ZmMADS36	FUL	AGAMOUS-like 8
	GRMZM2G113078	ZmEREB117	WRI1	Integrase-type DNA-binding superfamily protein
	GRMZM2G138976	ZmARID4	-	ARID/BRIGHT DNA-binding domain
	GRMZM5G842484	ZmHMG12	SSRP1	high mobility group
	GRMZM5G873335	-	-	ARID/BRIGHT DNA-binding domain
Kinases	GRMZM2G159678	-	-	Domain of unknown function (DUF303)
	GRMZM2G172900	-	NEK5	NIMA-related kinase 5
	GRMZM2G474546	-	-	Protein kinase superfamily protein
C. Yellow module – Highland landraces				
Transcription	AC186524.3_FG005	-	HMGB4	high mobility group B4
	GRMZM2G052667	ZmEREB102	HRE1	Integrase-type DNA-binding superfamily
	GRMZM2G098227	-	ARIA	ARM repeat protein interacting with ABF2
	GRMZM2G308034	ZmMYB46	MYB103	myb domain protein 103
Kinases	GRMZM2G126858	-	-	Leucine-rich repeat transmembrane protein kinase
	GRMZM2G139157	-	-	protein kinase superfamily protein
	GRMZM2G316907	-	-	Leucine-rich repeat protein kinase family
	GRMZM2G403719	-	CRK23	cysteine-rich RLK (RECEPTOR-like protein kinase) 23
D. Yellow module – Lowland landraces				
Transcription	GRMZM2G077197	-	NPR1	regulatory protein (NPR1)
	GRMZM2G327349	ZmWRKY99	WRKY42	WRKY family transcription factor
Kinases	GRMZM2G002542	-	-	kinase family protein w/leucine-rich repeat
	GRMZM2G119714	-	-	protein kinase superfamily protein
	GRMZM2G316474	-	-	Leucine-rich repeat protein kinase family

Functional category, broad categories defining function; Maize gene IDs, gene IDs from MaizeGDB; Maize TF; TF IDs from the Grassius GrassTFDB; TAIR symbol, gene symbol from The Arabidopsis Information Resource (TAIR); Arabidopsis TAIR10 definition, functional annotation from Phytozome; Hyphens, nonexistent data

#### Turquoise module ‘hub’ genes related to maximum normal mean temperature

We identified 155 ‘hub’ genes upon further investigation of the correlation between the turquoise module and maximum normal mean temperature (Additional file 10). Of these ‘hub’ genes, 133 (86%) were DE (FDR < 0.05) between highland and lowland maize landraces—59 were up-regulated in the highland landraces while 74 were up-regulated in lowland landraces (Additional file 10).

Six of the 59 ‘hub’ genes up-regulated in the highland landraces were functionally annotated as being transcription factors (TFs) or involved in transcriptional regulation (Table 2A). Nineteen of the remaining 53 ‘hub’ genes up-regulated in the highland landraces were functionally annotated as being involved in general and abiotic stress responses, mRNA stability, DNA repair, flowering time, carbon capture, the citric acid cycle, and kinase activity (Additional file 4).

Of the 74 ‘hub’ genes up-regulated in the lowland landraces, eight were functionally annotated as being TFs or influencing transcription (Table 2B). Twenty-seven of the remaining 66 ‘hub’ genes were functionally annotated as playing a role in hormone signaling, photosynthesis/respiration, abiotic stress, leaf shape/development, and kinase activity (Additional file 4).

#### Yellow module ‘hub’ genes related to minimum daily mean temperature

We identified 88 ‘hub’ genes upon inquiry into the yellow module – minimum daily mean temperature correlation (Additional file 10). Seventy-four of these 88 genes (84%) were DE (FDR < 0.05) between highland and lowland maize landraces along our study gradient. Of these 74 DE genes, 42 were up-regulated in the lowland landraces while 32 were up-regulated in those from the highlands.

Four of the 32 genes up-regulated in the highland landraces were TFs or interact with transcription factors (Table 2C). Eighteen of the remaining 28 ‘hub’ genes up-regulated in the highland landraces were functionally annotated as being involved in hormone signaling, RNA processing, abiotic/biotic stress, and kinase activity (Additional file 4).

Of the 42 ‘hub’ genes up-regulated in the lowland landraces, two were TFs (Table 2D). Twenty of the remaining 40 ‘hub’ genes up-regulated in the landraces from the lowlands were annotated as being involved in hormonal signaling, general and biotic stress, RNA editing, the citric acid cycle/electron transport chain, and kinase activity (Additional file 4).

## Discussion

### Novel RNA-seq based WGCNA approach at the landscape level

It is an essential evolutionary question to determine how genetic variation changes across diverse landscapes, thereby illuminating potential patterns of adaptation and the biological processes that underlie adaptation in plant populations. We sought to identify putative selective pressures along our study’s elevational and environmental gradient, while simultaneously determining the ways these pressures may have led to genetic differentiation along that gradient. To meet these objectives we combined a novel, common garden, transcriptomics approach with environmental parameter – module eigengene correlation analysis (WGCNA) at the landscape level. Elucidating strong environmental parameter – module correlations among populations can illuminate putative selective pressures potentially governing genetic differentiation across the landscape. Understanding of the potential biological functions of environmentally important modules can be gained through analysis of GO categories and ‘hub’ genes.

Here we report on genetic differentiation in maize landraces cultivated along an elevational gradient in Chiapas, Mexico. In particular, we demonstrated that the transcriptomes of the maize landraces from distinct elevations are differentiated (Figs. 1 and 4). The turquoise and yellow module expression values were highly correlated with maximum normal mean temperature and minimum daily mean temperature of landrace origin (Fig. 3), respectively, suggesting these environmental variables may have acted as selective pressures that shaped the transcriptomes. Overrepresented GO categories for these modules further suggest that environmental pressures, such as temperature, may indeed be responsible. Upon inquiring into the function of the maximum temperature-relevant turquoise module ‘hub’ genes and the minimum temperature-relevant yellow module ‘hub’ genes that were DE between highland and lowland landraces, we revealed that temperature may have selected for distinct, abiotic and biotic (respectively) stress coping mechanisms at different elevations (Additional file 4). In both modules, a number of transcription factors and kinases were identified as being ‘hub’ genes and deserve further attention as they may be key factors in the observed patterns of transcriptome differentiation (Table 2).

Yet signals of differential adaptation can be influenced by neutral processes [52], such as drift or isolation by distance. In maize, isolation by distance can be identified at around 50 km [5] or at shorter distances when gene flow is reduced by ethnolinguistic barriers to seed exchange [53] or other factors (e.g., flowering time differences) [17]. Thus, given the proximity of our elevations of origin and the ethnolinguistic diversity in the area, it is likely that some of the differentiation we have identified may have been influenced by neutral processes [52]. Nevertheless, our findings provide essential preliminary data on which to build further validation of putative adaptive genes.

### Differential adaptation of maize races

Elevation has shaped the transcriptomes of maize in Chiapas. In our study, landraces grouped by elevation of origin (Fig. 1), which was confounded with race with the exception of the *Olotillo* samples (landrace 20; Table 1). *Olotillo* is rarely cultivated in the highlands of Chiapas, which is reflected by the *Olotillo* transcriptome profiles being more similar to midland (*Comiteco*) landraces than were the rest of the highland landraces (Fig. 1). Interestingly, only portions of the *Olotillo* transcriptome followed this trend. Yellow module eigengene expression levels of the *Olotillo* landraces were more similar to those of the midland landraces, while *Olotillo* expression levels of the turquoise module were more similar to landraces from the highlands (Fig. 4). This suggests that a portion of the *Olotillo* genome may not be adapted to the highland environment and that there may be constraints on its evolution.

### GO categories of environmentally relevant modules indicative of stress responses

High temperature (or a factor highly correlated with it) may have selected for differentiation in plasma membrane repair mechanisms or environmental signaling in the maize landraces along our study gradient. Strong correlation between the turquoise module expression and maximum normal mean temperature of landrace origin (Fig. 3) pointed to enrichment of two GO categories involved in membrane vesicle trafficking (i.e., coated membrane/membrane coat; Additional file 9). The module itself and over 90% of the genes making up these GO categories, some of them clathrin-related, were up-regulated in the highland and midland landraces when compared to those from the lowlands (Fig. 4a, Additional file 11). Plasma membranes are essential to environmental signaling, as well as targets of injury during abiotic stress, since they directly interface with the outside environment [54].

Minimum temperature along our study gradient may have differentiated maize for environmentally relevant signaling cascades. Strong correlations between the yellow module expression and minimum temperature (Fig. 3) pointed to enrichment of several GO categories related to signal transduction, including calcium ion binding, protein tyrosine kinase activity, and protein serine/threonine kinase activity (Additional file 9). All of these can play a role in stress responses [55, 56].

### Temperature differentiates expression of genes related to hormones, abiotic stress response, and development

A number of DE ‘hub’ genes in the maximum temperature related turquoise module suggest that transcriptional differentiation has occurred in the maize landraces for genes associated with hormonal regulation, abiotic stress responses, and development. Highland and lowland maize landraces were differentiated for expression of genes related to hormone biosynthesis and signaling, which may represent distinct adaptations to maximum temperatures of landrace origin. The turquoise module (Fig. 3), contained a number of auxin-, abscisic acid- (ABA), and gibberellic acid- (GA)related genes. These hormone-related genes, such as YUC2, AAO3, HOS3, ABF4, GA1, GA2OX8, were up-regulated in the lowland landraces when compared to the highland landraces (Table 2B & Additional file 4). Thus, maximum temperature differences along our study gradient may have led to the genetic differentiation of hormone biosynthesis and signaling in the maize landraces.

Since genes making up co-expressed modules are assumed to be involved in the same biological function [30], other DE ‘hub’ genes in the turquoise module may provide information on how differential control of hormone biosynthesis and signaling in the maize landraces influenced physiological responses. For instance, the up-regulation of ‘hub’ genes known to affect leaf architecture (PLL4, PIP2, SPK1, ALE2) and contribute to abiotic stress responses (VTE1, GST30, SEP1, OSA1, and CBL10) in the lowland landraces (Additional file 4) may have been induced by increased levels of auxin, ABA, and/or alterations in GA levels in these landraces. In fact, leaf architecture ‘hub’ genes identified here have been reported as being responsive to auxin signals [57, 58] and plant hormones in general have been reported to partake in a number of abiotic stress responses [59–61].

Several ‘hub’ genes involved in responses to abiotic conditions and abiotic stress responses were identified in the turquoise module as highly correlated with maximum temperature and DE among elevations of landrace origin. The highland landraces exhibited up-regulation of a number of turquoise ‘hub’ genes indicative of responses to abiotic conditions (Additional file 4), including genes involved in photosynthesis (PPC3, c-NAD-MDH2) and several general stress related genes (FSD3-like, GRMZM2G005526; FSD3-like, GRMZM2G081585; SHM1; OTS1), which may indicate diverse stress responses. We also found up-regulation in the lowland landraces of multiple ‘hub’ genes involved in cellular respiration (GAMMA CA1, ETFALPHA, UCP1, and COX2) (Additional file 4).

Maximum temperature where maize landraces originated may have also differentiated development, such as flowering time. We found TFs and genes related to the regulation of developmental transitions that were ‘hub’ genes in the turquoise module and were DE between highland and lowland landraces. The lowland landraces were up-regulated for ZmSBP29, FUL, and a SSRP1-like gene, while the highlands were up-regulated for ZmSBP27 and a VRN5/VIL1-like gene (Tables 2 & Additional file 4: Table S4), all of which are relevant to developmental phase changes and flowering. In sum, conditions in the lowlands and highlands may have differentially selected upon gene expression controlling a range of traits, from hormonal signaling to flowering time, that can affect interactions with abiotic factors.

### Temperature induced differences in genes related to hormone signaling in biotic stress responses

The nature of many of the ‘hub’ genes in the minimum temperature related yellow module (Fig. 3) suggest that hormone signaling related to biotic stress responses was differentiated among landraces of maize from high and low elevations. Hormones play an important role in biotic stress responses [62–64]. Expression of genes related to hormones involved in biotic responses were differentiated between highland and lowland landraces. For instance, highland landraces were up-regulated for the jasmonic acid-related ‘hub’ gene, OPR1, and two ABA-related genes (CPK32, ARIA), as well as five genes related to the hormonal regulation of pathogen response (LAP2, LAZ1, VAD1, MOS1, and CNGC2) (Table 2 & Additional file 4). Similarly, we found at least four other ‘hub’ genes, up-regulated in the lowland landrace, that indicated differentiation in hormone-induced responses to pathogens (RPS2, RPS3, WAK3, WAK5, and ACD11) (Additional file 4). Additionally, the lowland landraces showed up-regulation of ‘hub’ genes involved in the biosynthesis and signaling of phytosterols and salicylic acid, including CAS1, NPR1, and RLK (Table 2 & Additional file 4). Thus, different environmental conditions in the lowlands and highlands may have led to differential selection by pathogens and, therefore, expression of genes involved in biotic stress responses.

### Transcription factors and kinases—Targets for further study

A range of DE TFs and kinase ‘hub’ genes were found in the turquoise and yellow modules (Table 2). These genes may play central roles in the apparent temperature induced differentiation of biotic and abiotic stress responses in the maize landraces of Chiapas. Since genes making up co-expressed modules are assumed to be co-regulated and involved in the same biological function [65], transcription factors and kinases within modules can play important roles in regulating those functions and signaling cascades. The eight unannotated turquoise module TF ‘hub’ genes – four up-regulated in the highland landraces (ZmGLK18, GRMZM2G154169, GRMZM2G414141, ZmbHLH81) and four in the lowland landraces (ZAT10, ZmEREB117, ZmARID4, GRMZM5G873335) (Table 2) – may regulate key aspects of the abiotic stress response differences observed between the highland and lowland landraces. Likewise, three unannotated yellow module transcription factor ‘hub’ genes up-regulated in the highland landraces (HMGB4, ZmEREB102, and ZmMYB46), along with the one up-regulated in the lowland landraces (ZmWRKY99), may play regulatory roles in the apparently different biotic stress responses. Similarly, the additional turquoise and yellow module kinases (Table 2) may have been involved in the differential abiotic and biotic stress response. Given the apparent ‘cross-talk’ between abiotic and biotic stress responses [66], there may also be interesting relationships between these regulator genes.

## Conclusions

Here, we have presented a novel approach of using RNA-seq in combination with WCGNA that can be employed to understand how genetic diversity shaped by natural selection is distributed across the landscape. We have shown that the transcriptomes of maize landraces spanning an elevational gradient in Chiapas, Mexico are differentiated according to elevation of landrace origin. Upon further inquiry, we identified two co-expression modules that were associated with temperature related parameters of landrace origin. As we might expect, temperature appears to be an important selective pressure in Chiapas that likely led to the differentiation in hormone biosynthesis/signaling and subsequent abiotic and biotic stress responses in the maize landraces. Among the ‘hub’ genes identified in each module were a number of transcription factors and kinases, some as yet unannotated, that may be involved in regulating and signaling the apparent abiotic and biotic stress responses, respectively. Hypothesis-driven studies looking at the role of these transcription factors along with physiological studies aimed at better understanding the precise mechanisms and selective pressures responsible for the apparent genetic differentiation would enhance our understanding of local adaptation in the maize landraces of Chiapas.

## Additional files

Additional file 1: Weather station information nearest each landrace. (DOC 24 kb)

Additional file 2: Environmental parameters definitions. (DOC 23 kb)

Additional file 3 Hierarchical clustering of co-expressed modules. (DOC 869 kb)

Additional file 4: ‘Hub’ genes of interest other than transcription factors and kinases [67–85]. (DOC 82 kb)

Additional file 5: Raw, trimmed and mapped read counts for each of the 45 maize landrace RNA-seq libraries. (DOC 89 kb)

Additional file 6: Thirty year precipitation (A) and evaporation (B) related environmental parameters of maize landrace origin. (DOC 514 kb)

Additional file 7: Environmental variation due to landscape variation. (DOC 22 kb)

Additional file 8: Co-expression module gene counts. (DOC 43 kb)

Additional file 9: GO enrichment analysis for the turquoise (A) and yellow (B) co-expression modules. (DOC 41 kb)

Additional file 10: Hub genes. (DOC 331 kb)

Additional file 11: Genes making up the turquoise module overrepresented GO categories coated membrane and membrane coat. (DOC 42 kb)

## Abbreviations

ABA: Abscisic acid
ATP: Adenosine triphosphate
bp: Base pairs
DE: Differentially expressed
FDR: False discovery rate
GA: Gibberellic acid
GO: Gene ontology
GS: Gene significance
MCBL: Molecular and Cellular Imaging Center Computation Biology Laboratory
MM: Module membership
OARDC: Ohio Agricultural Research and Development Center
PCA: Principal component analysis
RNA: Ribonucleic acid
RNA-seq: RNA sequencing
s.d.: Standard deviation
TAIR: The Arabidopsis Information Resource
TFs: Transcription factors
UVB: Ultraviolet B
VST: Variance stabilized transformed
WGCNA: Weighted gene co-expression network analysis

## References

[1] Matsuoka Y, Vigouroux Y, Goodman MM, Sanchez J, Buckler E, Doebley J (2002). A single domestication for maize shown by multilocus microsatellite genotyping. PNAS.

[2] Piperno DR, Ranere AJ, Holst I, Iriarte J, Dickau R (2009). Starch grain and phytolith evidence for early ninth millennium B.P. Maize from the central Balsas River valley, Mexico. PNAS.

[3] van Heerwaarden J, Doebley J, Briggs WH, Glaubitz JC, Goodman MM, Gonzalez JDJS (2011). Genetic signals of origin, spread, and introgression in a large sample of maize landraces. PNAS.

[4] Sanchez JJ, Goodman MM, Stuber CW (2000). Isozymatic and morphological diversity in the races of maize of Mexico. Econ Bot.

[5] Vigouroux Y, Glaubitz JC, Matsuoka Y, Goodman MM, Sánchez GJ, Doebley J (2008). Population structure and genetic diversity of new world maize races assessed by DNA microsatellites. Am J Bot.

[6] Vavilov NI. Origin and geography of cultivated plants. New York: Cambridge University Press; 1992.

[7] Harlan JR. Crops and man. Madison: American Society of Agronomy; 1975. p. 306 pp.

[8] Wilkes HG (1977). Hybridization of maize and teosinte, in Mexico and Guatemala and the improvement of maize. Econ Bot.

[9] Doebley J (2004). The genetics of maize evolution. Annu Rev Genet.

[10] Ruiz Corral JA, Durán Puga N, Sánchez González JDJ, Ron Parra J, González Eguiarte DR, Holland JB (2008). Climatic adaptation and ecological descriptors of 42 Mexican maize races. Crop Sci.

[11] Ureta C, González-Salazar C, González EJ, Álvarez-Buylla ER, Martínez-Meyer E (2013). Environmental and social factors account for Mexican maize richness and distribution: a data mining approach. Agric Ecosyst Environ.

[12] Brush SB (1995). In situ conservation of landraces in centers of crop diversity. Crop Sci.

[13] Wellhausen EJ, Roberts LM, Hernandez XE, Mangelsdorf PC. Races of maize in Mexico. Their origin, characteristics and distribution. 1952. p. 223 pp.

[14] Perales HR, Hernández-Casillas JM (2005). Diversidad del maíz en Chiapas.

[15] Brush SB, Perales HR (2007). A maize landscape: ethnicity and agro-biodiversity in Chiapas Mexico. Agric Ecosyst Environ.

[16] van Etten J, López MRF, Monterroso LGM, Samayoa KMP (2008). Genetic diversity of maize (Zea Mays L. Ssp. Mays) in communities of the western highlands of Guatemala: geographical patterns and processes. Genet Resour Crop Evol.

[17] Pressoir G, Berthaud J (2004). Patterns of population structure in maize landraces from the central valleys of Oaxaca in Mexico. Heredity.

[18] Pressoir G, Berthaud J (2004). Population structure and strong divergent selection shape phenotypic diversification in maize landraces. Heredity.

[19] Perales HR, Benz BF, Brush SB (2005). Maize diversity and ethnolinguistic diversity in Chiapas, Mexico. PNAS.

[20] Benz B, Perales H, Brush S (2007). Tzeltal and Tzotzil farmer knowledge and maize diversity in Chiapas, Mexico. Curr Anthropol.

[21] van Etten J, de Bruin S (2007). Regional and local maize seed exchange and replacement in the western highlands of Guatemala. Plant Genet Resour.

[22] Mercer K, Martínez-Vásquez Á, Perales HR (2008). Asymmetrical local adaptation of maize landraces along an altitudinal gradient. Evol Appl.

[23] Holliday JA, Ralph SG, White R, Bohlmann J, Aitken SN (2008). Global monitoring of autumn gene expression within and among phenotypically divergent populations of Sitka spruce (Picea Sitchensis). New Phytol.

[24] Hayano-Kanashiro C, Calderón-Vázquez C, Ibarra-Laclette E, Herrera-Estrella L, Simpson J (2009). Analysis of gene expression and physiological responses in three Mexican maize landraces under drought stress and recovery irrigation. PLoS One.

[25] Schoville SD, Barreto FS, Moy GW, Wolff A, Burton RS (2012). Investigating the molecular basis of local adaptation to thermal stress: population differences in gene expression across the transcriptome of the copepod Tigriopus Californicus. BMC Evol Biol.

[26] Lenz TL, Eizaguirre C, Rotter B, Kalbe M, Milinski M (2013). Exploring local immunological adaptation of two stickleback ecotypes by experimental infection and transcriptome-wide digital gene expression analysis. Mol Ecol.

[27] Manousaki T, Hull PM, Kusche H, Machado-Schiaffino G, Franchini P, Harrod C (2013). Parsing parallel evolution: ecological divergence and differential gene expression in the adaptive radiations of thick-lipped Midas cichlid fishes from Nicaragua. Mol Ecol.

[28] Raney JA, Reynolds DJ, Elzinga DB, Page J, Udall JA, Jellen EN (2014). Transcriptome analysis of drought induced stress in Chenopodium Quinoa. Am J Plant Sci.

[29] Zhang B, Horvath S (2005). A general framework for weighted gene co-expression network analysis. Stat Appl Genet Molec Biol.

[30] Wolfe CJ, Kohane IS, Butte AJ (2005). Systematic survey reveals general applicability of “guilt-by-association” within gene coexpression networks. BMC Bioinformatics..

[31] Oldham MC, Horvath S, Geschwind DH (2006). Conservation and evolution of gene coexpression networks in human and chimpanzee brains. PNAS.

[32] Filteau M, Pavey SA, St-Cyr J, Bernatchez L (2013). Gene coexpression networks reveal key drivers of phenotypic divergence in lake whitefish. Mol Biol Evol.

[33] Munkvold JD, Laudencia-Chingcuanco D, Sorrells ME (2013). Systems genetics of environmental response in the mature wheat embryo. Genetics.

[34] Cañas RA, Canales J, Muñoz-Hernández C, Granados JM, Ávila C, García-Martín ML (2015). Understanding developmental and adaptive cues in pine through metabolite profiling and co-expression network analysis. J Exp Bot.

[35] Breedlove DE (1973). The phytogeography and vegetation of Chiapas (Mexico).

[36] Zhong S, Joung J-G, Zheng Y, Chen Y, Liu B, Shao Y (2011). High-throughput illumina strand-specific RNA sequencing library preparation. Cold Spring Harb Protoc.

[37] Giardine B, Riemer C, Hardison RC, Burhans R, Elnitski L, Shah P (2005). Galaxy: a platform for interactive large-scale genome analysis. Genome Res.

[38] Andrews S. FastQC: a quality control tool for high throughput sequence data. Reference Source. 2010. Available from: http://www.bioinformatics.babraham.ac.uk/projects/fastqc.

[39] Martin M (2011). Cutadapt removes adapter sequences from high-throughput sequencing reads. EMBnet J.

[40] Kofler R, Orozco-terWengel P, De Maio N, Pandey RV, Nolte V, Futschik A (2011). PoPoolation: a toolbox for population genetic analysis of next generation sequencing data from pooled individuals. PLoS One.

[41] Goodstein DM, Shu S, Howson R, Neupane R, Hayes RD, Fazo J (2012). Phytozome: a comparative platform for green plant genomics. Nucl Acids Res..

[42] Kim D, Pertea G, Trapnell C, Pimentel H, Kelley R, Salzberg SL (2013). TopHat2: accurate alignment of transcriptomes in the presence of insertions, deletions and gene fusions. Genome Biol.

[43] Anders S, Pyl PT, Huber W. HTSeq - A Python framework to work with high-throughput sequencing data. Bioinformatics. 2015;31:166–9. 10.1093/bioinformatics/btu638 PMC4287950 25260700

[44] Anders S, Huber W (2010). Differential expression analysis for sequence count data. Genome Biol.

[45] Langfelder P, Horvath S (2008). WGCNA: an R package for weighted correlation network analysis. BMC Bioinformatics.

[46] Fuller TF, Ghazalpour A, Aten JE, Drake TA, Lusis AJ, Horvath S (2007). Weighted gene coexpression network analysis strategies applied to mouse weight. Mamm Genome.

[47] Lawrence CJ, Seigfried TE, Brendel V (2005). The maize genetics and genomics database. The community resource for access to diverse maize data. Plant Physiol.

[48] Rhee SY, Beavis W, Berardini TZ, Chen G, Dixon D, Doyle A (2003). The Arabidopsis information resource (TAIR): a model organism database providing a centralized, curated gateway to Arabidopsis biology, research materials and community. Nucl Acids Res.

[49] Gray J, Bevan M, Brutnell T, Buell CR, Cone K, Hake S (2009). A recommendation for naming transcription factor proteins in the grasses. Plant Physiol.

[50] Yilmaz A, Nishiyama MY, Fuentes BG, Souza GM, Janies D, Gray J (2009). GRASSIUS: a platform for comparative regulatory genomics across the grasses. Plant Physiol.

[51] Langfelder P, Horvath S (2007). Eigengene networks for studying the relationships between co-expression modules. BMC Syst Biol.

[52] Pavlidis P, Jensen JD, Stephan W, Stamatakis A (2012). A critical assessment of storytelling: gene ontology categories and the importance of validating genomic scans. Mol Biol Evol.

[53] Orozco-Ramirez Q, Ross-Ibarra J, Santacruz-Varela A, Brush S (2016). Maize diversity associated with social origin and environmental variation in southern Mexico. Heredity.

[54] Takahashi D, Li B, Nakayama T, Kawamura Y, Uemura M (2013). Plant plasma membrane proteomics for improving cold tolerance. Front Plant Sci.

[55] Ghelis T, Bolbach G, Clodic G, Habricot Y, Miginiac E, Sotta B (2008). Protein tyrosine kinases and protein tyrosine phosphatases are involved in abscisic acid-dependent processes in Arabidopsis seeds and suspension cells. Plant Physiol.

[56] Ghelis T (2011). Signal processing by protein tyrosine phosphorylation in plants. Plant Signal Behav.

[57] Paciorek T, Zažímalová E, Ruthardt N, Petrášek J, Stierhof Y-D, Kleine-Vehn J (2005). Auxin inhibits endocytosis and promotes its own efflux from cells. Nature.

[58] Lin D, Nagawa S, Chen J, Cao L, Chen X, Xu T (2012). A ROP GTPase-dependent auxin signaling pathway regulates the subcellular distribution of PIN2 in Arabidopsis roots. Curr Biol.

[59] Nakashima K, Yamaguchi-Shinozaki K, Shinozaki K (2014). The transcriptional regulatory network in the drought response and its crosstalk in abiotic stress responses including drought, cold, and heat. Front Plant Sci.

[60] Peleg Z, Blumwald E (2011). Hormone balance and abiotic stress tolerance in crop plants. Curr Opin Plant Biol.

[61] Colebrook EH, Thomas SG, Phillips AL, Hedden P (2014). The role of gibberellin signalling in plant responses to abiotic stress. J Exp Biol.

[62] Kunkel BN, Brooks DM (2002). Cross talk between signaling pathways in pathogen defense. Curr Opin Plant Biol.

[63] Vos IA, Verhage A, Schuurink RC, Watt LG, Pieterse CMJ, Van Wees SCM (2013). Onset of herbivore-induced resistance in systemic tissue primed for jasmonate-dependent defenses is activated by abscisic acid. Front Plant Sci.

[64] Durner J, Shah J, Klessig DF (1997). Salicylic acid and disease resistance in plants. Trends Plant Sci.

[65] Moreno-Risueno MA, Busch W, Benfey PN (2010). Omics meet networks — using systems approaches to infer regulatory networks in plants. Curr Opin Plant Biol.

[66] Fujita M, Fujita Y, Noutoshi Y, Takahashi F, Narusaka Y, Yamaguchi-Shinozaki K (2006). Crosstalk between abiotic and biotic stress responses: a current view from the points of convergence in the stress signaling networks. Curr Opin Plant Biol.

[67] Myouga F, Hosoda C, Umezawa T, Iizumi H, Kuromori T, Motohashi R (2008). A heterocomplex of iron superoxide dismutases defends chloroplast nucleoids against oxidative stress and is essential for chloroplast development in Arabidopsis. Plant Cell.

[68] Simon C, Langlois-Meurinne M, Didierlaurent L, Chaouch S, Bellvert F, Massoud K (2014). The secondary metabolism glycosyltransferases UGT73B3 and UGT73B5 are components of redox status in resistance of Arabidopsis to pseudomonas syringae pv. Tomato. Plant Cell Environ.

[69] Edwards G, Walker DA (1983). C3, C4: mechanisms, and cellular and environmental regulation, of photosynthesis.

[70] Quist TM, Sokolchik I, Shi H, Joly RJ, Bressan RA, Maggio A (2009). HOS3, an ELO-like gene, inhibits effects of ABA and implicates a S-1-P/ceramide control system for abiotic stress responses in Arabidopsis Thaliana. Mol Plant.

[71] Schomburg FM, Bizzell CM, Lee DJ, Zeevaart JAD, Amasino RM (2003). Overexpression of a novel class of Gibberellin 2-Oxidases decreases Gibberellin levels and creates dwarf plants. Plant Cell.

[72] Steinum TM, Berner HS (1998). Stacy R a. P, Salehian Z, Aalen RB. Differential regulation of the barley (Hordeum Vulgare) transcripts B22E and B12D in mature aleurone layers. Physiol Plant.

[73] Mulo P (1807). Chloroplast-targeted ferredoxin-NADP+ oxidoreductase (FNR): structure, function and location. BBA Bioenergetics.

[74] Capaldi RA (1990). Structure and function of cytochrome c oxidase. Annu Rev Biochem.

[75] Jasinski M, Sudre D, Schansker G, Schellenberg M, Constant S, Martinoia E (2008). AtOSA1, a member of the Abc1-like family, as a new factor in cadmium and oxidative stress response. Plant Physiol.

[76] Kalyna M, Lopato S, Barta A (2003). Ectopic expression of atRSZ33 reveals its function in splicing and causes pleiotropic changes in development. Mol Biol Cell.

[77] Wang W, Vinocur B, Shoseyov O, Altman A (2004). Role of plant heat-shock proteins and molecular chaperones in the abiotic stress response. Trends Plant Sci.

[78] De Angeli A, Zhang J, Meyer S, Martinoia E (2013). AtALMT9 is a malate-activated vacuolar chloride channel required for stomatal opening in Arabidopsis. Nat Commun.

[79] Malinovsky FG, Brodersen P, Fiil BK, McKinney LV, Thorgrimsen S, Beck M (2010). Lazarus1, a DUF300 protein, contributes to programmed cell death associated with Arabidopsis acd11 and the hypersensitive response. PLoS One.

[80] Crouzet J, Trombik T, Fraysse ÅS, Boutry M (2006). Organization and function of the plant pleiotropic drug resistance ABC transporter family. FEBS Lett.

[81] He Z-H, He D, Kohorn BD (1998). Requirement for the induced expression of a cell wall associated receptor kinase for survival during the pathogen response. Plant J.

[82] He Z-H, Cheeseman I, He D, Kohorn BD (1999). A cluster of five cell wall-associated receptor kinase genes, Wak1–5, are expressed in specific organs of Arabidopsis. Plant Mol Biol.

[83] Sivaguru M, Ezaki B, He Z-H, Tong H, Osawa H, Baluška F (2003). Aluminum-induced gene expression and protein localization of a cell wall-associated receptor kinase in Arabidopsis. Plant Physiol.

[84] Walker JE (1992). The NADH:ubiquinone oxidoreductase (complex I) of respiratory chains. Q Rev Biophys.

[85] León G, Holuigue L, Jordana X (2007). Mitochondrial complex II is essential for gametophyte development in Arabidopsis. Plant Physiol.

