# Supplementary material for: Differentiated transcriptional signatures in the maize landraces of Chiapas, Mexico
Source: BMC Genomics. 2017 Sep 8;18:707. doi: 10.1186/s12864-017-4005-y (PMC5591509; doi:10.1186/s12864-017-4005-y)
Supplement: Supplementary file 1 — Weather station information nearest each landrace. (DOC 24 kb) [file 12864_2017_4005_MOESM1_ESM.doc]

Additional files

Additional file 1: Weather station information nearest each landrace

**00007224** (Chicomuselo, Chicomuselo): Landraces 1, 4 & 6

Distance from station

Landrace 1 – 15.95 km (9.91 miles)

Landrace 4 – 10.85 km (6.74 miles)

Landrace 6 – 10.85 km (6.74 miles)

**Station 00007009** (Aquespala, F. Comalapa): Landraces 7 & 9

Distance from station

Landrace 7 – 7.06 km (4.38 miles)

Landrace 9 – 5.27 km (3.27 miles)

**Station 00007231** (Tziscao, La Trinitaria): Landraces 10 & 12

Landrace 10 – 8.51 km (5.29 miles)

Landrace 12 – 12.15 km (7.55 miles)

**Station 00007205** (Comitan, Comitan): Landrace 13

Landrace 13 – 3.37 km (2.10 miles)

**Station 00007104** (Las Margaritas, Las Margaritas): Landraces 17 & 18

Landrace 17 – 7.45 km (4.63 miles)

Landrace 18 – 7.45 km (4.63 miles)

**Station 00007002** (Abelardo Rodriquez, Comitan): Landrace 20

Landrace 20 – 6.53 km (4.06 miles)

**Station 00007087** (La Cabana, S.C. Las Casas): Landraces 26, 27, 29 & 30

Landrace 26 – 5.48 km (3.41 miles)

Landrace 27 – 5.72 km (3.56 miles)

Landrace 29 – 14.58 km (9.06 miles)

Landrace 30 – 14.51 km (9.02 miles)

Weather station data nearest each of the landraces used in our analyses. Data from these stations were used to generate Fig. 2 and were also used in the module – environmental parameter correlation analysis. Thirty year averages (1971-2000) for each station was obtained from Comisión Nacional del Agua (CONAGUA) – Servicio Meteorológico Nacional. México. (http://www.smn.cna.gob.mx/). The distance each landrace was away from its nearest weather station is provided in both kilometers (km) and miles.
