# Supplementary material for: Differentiated transcriptional signatures in the maize landraces of Chiapas, Mexico
Source: BMC Genomics. 2017 Sep 8;18:707. doi: 10.1186/s12864-017-4005-y (PMC5591509; doi:10.1186/s12864-017-4005-y)
Supplement: Supplementary file 2 — Environmental parameters definitions. (DOC 23 kb) [file 12864_2017_4005_MOESM2_ESM.doc]

Additional file 2: Environmental parameters definitions

Thirty year normal temperature, precipitation, and evaporation related environmental parameters of the 15 maize landraces used in the study were obtained from weather stations nearest each population (data obtained from; CONAGUA http://www.smn.cna.gob.mx/). Max_NM_Temp (*maximum normal mean temperature*) – Average daily maximum temperatures for each month averaged over 30 years; Max_MM_Temp (*maximum monthly mean temperature*) – Highest average of daily maximum temperatures recorded for each month over 30 years; Max_DM_Temp (*maximum daily mean temperature*) – Highest daily maximum temperatures recorded in a given month during 30 years; Normal_M_Temp (*normal mean temperature*) – Mean temperatures for each month averaged over 30 years; Min_NM_Temp (*minimum normal mean temperature)* – Average daily minimum temperatures for each month averaged over 30 years; Min_MM_Temp (*minimum monthly mean temperature*) – Lowest average of daily minimum temperatures recorded for a given month over 30 years; Min_DM_Temp (*minimum daily mean temperature*) – Lowest daily minimum temperatures recorded in a given month during 30 years; Normal_M_Prec (*Normal mean precipitation*) – Mean precipitation for each month averaged over 30 years; Max_MM_Prec (*Maximum monthly mean precipitation*) – Maximum monthly rainfall for each month over 30 years; Max_DM_Prec (*Maximum daily mean precipitation*) – Maximum rainfall amount in a given day for each month during 30 years; Normal_M_Evapo (*Normal mean evaporation*) – Mean evaporation for each month averaged over 30 years. Each environmental parameter was then averaged over all 12 months in a year.
