# Supplementary material for: Differentiated transcriptional signatures in the maize landraces of Chiapas, Mexico
Source: BMC Genomics. 2017 Sep 8;18:707. doi: 10.1186/s12864-017-4005-y (PMC5591509; doi:10.1186/s12864-017-4005-y)
Supplement: Supplementary file 3 — Hierarchical clustering of co-expressed modules. (DOC 869 kb) [file 12864_2017_4005_MOESM3_ESM.doc]

Additional file 3: Hierarchical clustering of co-expressed modules


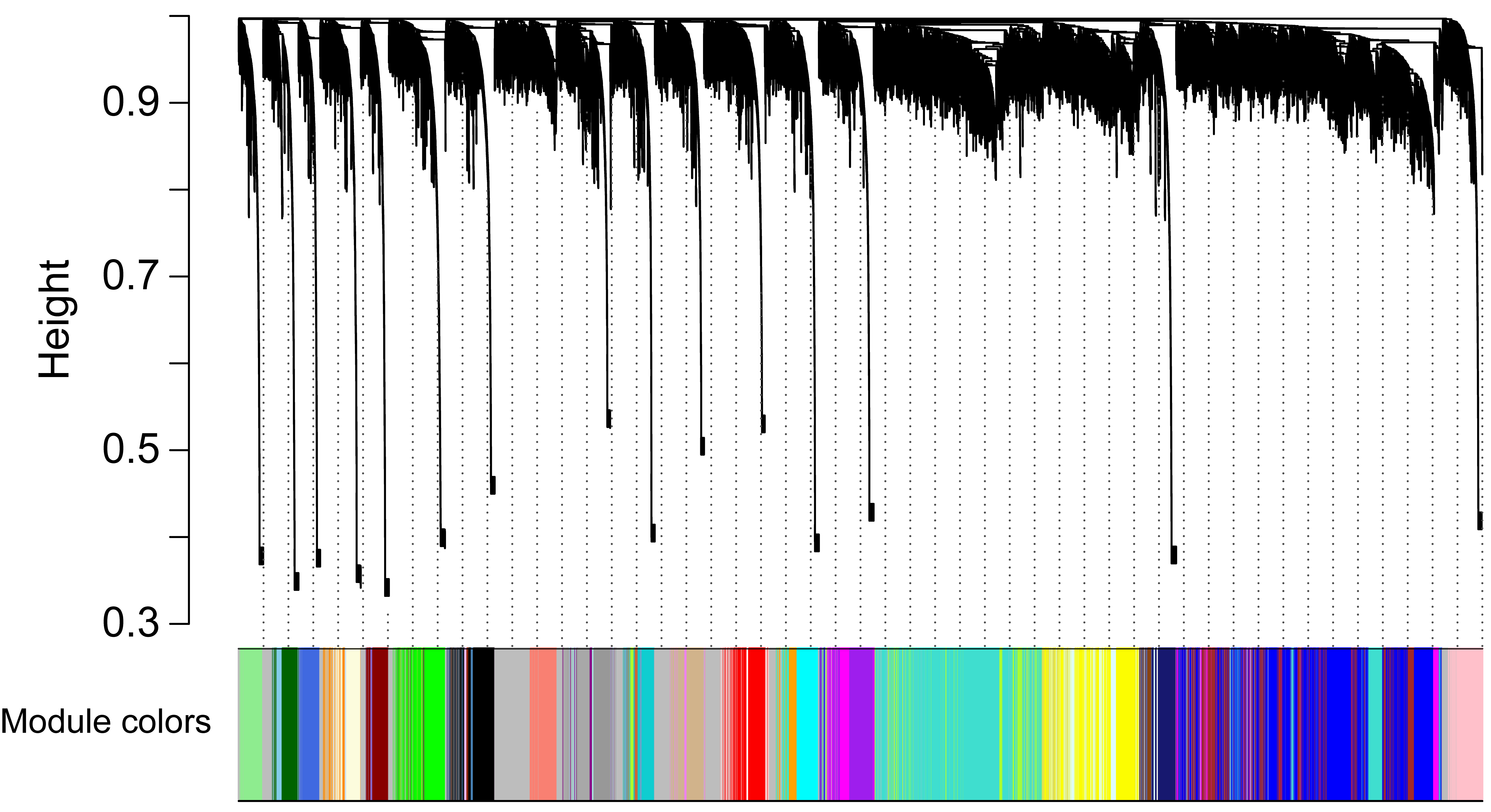


The dendrogram was generated using topological overlap dissimilarity measures (1-TOM) to identify distinct co-expression modules. Topological overlap matrices (TOM) measure how interconnected or similar two genes (nodes) are to each other. Branches correspond to co-expression modules as do module colors on the x-axis. 44 modules were identified. Height on the y-axis represents distance based on dissimilarity—lower values corresponding to the most connected genes in a given module [29].
