# Supplementary material for: Differentiated transcriptional signatures in the maize landraces of Chiapas, Mexico
Source: BMC Genomics. 2017 Sep 8;18:707. doi: 10.1186/s12864-017-4005-y (PMC5591509; doi:10.1186/s12864-017-4005-y)
Supplement: Supplementary file 4 — ‘Hub’ genes of interest other than transcription factors and kinases [67–85]. (DOC 82 kb) [file 12864_2017_4005_MOESM4_ESM.doc]

Additional file 4: ‘Hub’ genes of interest other than transcription factors and kinases. Turquoise module ‘hub’ genes upregulated in the highland (A) and lowland (B) landraces; yellow module ‘hub’ genes upregulated in the highland (C) and lowland (D) landraces organized by functional categories

| **A. Turquoise module – Highland landraces** | | | |  | | |  |
| --- | --- | --- | --- | --- | --- | --- | --- |
| **Functional category** | **Maize gene ID** | **MaizeTF** | | | **TAIR symbol** | | **Arabidopsis TAIR10 definition** |
| **General stress** | GRMZM2G005526 | **-** | | | FSD3 | | Fe superoxide dismutase 3 [67] |
|  | GRMZM2G081585 | **-** | | | FSD3 | | Fe superoxide dismutase 3 [67] |
|  | GRMZM2G135283 | **-** | | | SHM1 | | Serine transhydroxymethyltransferase 1 |
|  | GRMZM2G351786 | **-** | | | OTS1 | | UB-like protease 1D |
| **Biotic stress** | GRMZM2G349565 | **-** | | | **-** | | LRR and NB-ARC domains-containing disease resistance protein |
|  | GRMZM2G439784 | **-** | | | **-** | | disease resistance family protein / LRR family |
|  | GRMZM2G074631 | **-** | | | UGT73B3 | | UDP-glucosyl transferase 73B3 [68] |
| **mRNA stability** | GRMZM2G449779 | **-** | | | PUM23 | | Pumilio 23 |
| **DNA repair** | GRMZM2G003225 | **-** | | | NSE4 | | Nse4 component of Smc5/6 DNA repair complex |
| **Flowering time** | GRMZM2G021471 | **-** | | | VRN5/VIL1 | | Fibronectin type III domain-containing protein |
| **Carbon capture** | GRMZM2G074122 | **-** | | | PPC3 | | Phosphoenolpyruvate carboxylase 3 [69] |
| **Citric acid cycle** | GRMZM2G101290 | **-** | | | c-NAD-MDH2 | | Lactate/malate dehydrogenase family protein |
| **B. Turquoise module – Lowland landraces** | | | | | | |  |
| **Hormone signaling:** *Auxin* | GRMZM2G017193 | **-** | | | YUC2 | | Flavin-binding monooxygenase family protein |
|  | GRMZM2G064371 | **-** | | | ABP | | Endoplasmic reticulum auxin binding protein 1 |
|  | GRMZM2G078508 | **-** | | | ABP | | Endoplasmic reticulum auxin binding protein 1 |
|  | GRMZM2G435393 | **-** | | | CHS | | Chalcone and stilbene synthase family protein |
| **Hormone signaling:** *ABA* | GRMZM2G124175 | | **-** | | | AAO3 | Abscisic aldehyde oxidase 3 |
|  | GRMZM2G133793 | | **-** | | | HOS3-1 | GNS1/SUR4 membrane protein family [70] |
| **Hormone signaling:** *GA* | GRMZM2G006964 | |  | | | GA2OX8 | Gibberellin 2-oxidase 8 [71] |
|  | GRMZM2G068808 | | **-** | | | GA1 | Terpenoid cyclases/Protein prenyltransferases superfamily |
|  | GRMZM2G103617 | | **-** | | | B12D | B12D protein [72] |
| **Photosynthesis/Respiration** | GRMZM2G046924 | | **-** | | | GAMMA CA1 | Gamma carbonic anhydrase 1 |
|  | GRMZM2G178756 | | **-** | | | PEPKR1 | PEP carboxylase-relate kinase 1 |
|  | GRMZM2G320305 | | **-** | | | FNR2 | Ferredoxin-NADP(+)-oxidoreductase 2 [73] |
|  | GRMZM2G430526 | | **-** | | | ETFALPHA | Electron transfer flavoprotein alpha |
|  | GRMZM5G852877 | | **-** | | | UCP1 | Plant uncoupling mitochondrial protein 1 |
|  | GRMZM5G862955 | | **-** | | | COX2 | Cytochrome oxidase 2 [74] |
| **Abiotic stress** | GRMZM2G009785 | | **-** | | | VTE1 | Tocopherol cyclase, chloroplast/vitamin E deficient 1 (VTE1/SXD1) |
|  | GRMZM2G077206 | | **-** | | | GST30 | Glutathione S-transferase family protein |
|  | GRMZM2G080355 | | **-** | | | SEP1 | Stress enhanced protein 1 |
|  | GRMZM2G088375 | | **-** | | | OSA1 | ABC2 homolog 13 [75] |
|  | GRMZM2G116584 | | **-** | | | CBL10 | Calcineurin B-like protein 10 |
| **B. Turquoise module – Lowland landraces cont.** | | | | | |  |  |
| **Functional category** | **Maize gene ID** | | **MaizeTF** | | | **TAIR symbol** | **Arabidopsis TAIR10 definition** |
| **Leaf shape/development** | GRMZM2G093776 | | **-** | | | PLL4 | Poltergeist like 4 |
|  | GRMZM2G178693 | | **-** | | | PIP2 | Plasma membrane intrinsic protein 2 |
|  | GRMZM2G422537 | | **-** | | | SPK1 | Guanyl-nucleotide exchange factors; GTPase binding |
|  | GRMZM5G871520 | | **-** | | | ALE2 | Protein kinase superfamily protein |
| **C. Yellow module – Highland landraces** | | | | | |  |  |
| **Hormone signaling:** *ABA* | GRMZM2G003059 | | **-** | | | CPK32 | Calcium-dependent protein kinase 24 |
| **Hormone signaling:** *JA* | GRMZM2G068947 | | **-** | | | OPR1 | 12-oxophytodienoate reductase 1 |
| **RNA processing** | GRMZM2G060160 | | **-** | | | **-** | Tetratricopeptide repeat (TPR)-like superfamily |
|  | GRMZM2G065284 | | **-** | | | RSZ33 | Arginine/serine-rich zinc knuckle protein 33 [76] |
|  | GRMZM2G416498 | | **-** | | | RPF3 | Tetratricopeptide repeat (TPR)-like |
|  | GRMZM2G459702 | | **-** | | | **-** | Pseudouridine synthase family protein |
| **General stress** | GRMZM2G018558 | | **-** | | | LAP2 | Cytosol aminopeptidase family protein |
| **Abiotic stress** | GRMZM2G039886 | | **-** | | | HSP40 | DNAJ heat shock family protein [77] |
|  | GRMZM2G057983 | | **-** | | | ALMT9 | Aluminum-activated malate transporter 9 [78] |
|  | GRMZM2G428391 | | **-** | | | HSP70 | Heat shock protein 70 [77] |
| **Biotic stress** | GRMZM2G088737 | | **-** | | | LAZ1 | [79] |
|  | GRMZM2G158248 | | **-** | | | VAD1 | GRAM domain family protein |
|  | GRMZM2G418515 | | **-** | | | MOS1 | Modifier of snc1 |
|  | GRMZM5G858887 | | **-** | | | CNGC2 | Cyclic nucleotide-regulated ion channel family protein |
| **D. Yellow module – Lowland landraces** | | | | | |  |  |
| **Hormone signaling:** *BRs* | GRMZM2G070575 | | **-** | | | CAS1 | Cycloartenol synthase 1 |
| **Hormone signaling:** *SA* | GRMZM2G475948 | | **-** | | | RLK | Receptor lectin kinase |
| **General stress** | GRMZM2G043857 | | **-** | | | **-** | Ankyrin repeat family protein |
|  | GRMZM2G118243 | | **-** | | | PDR4 | Pleiotropic drug resistance 4 [80] |
|  | GRMZM2G121024 | | **-** | | | **-** | Heavy metal transport/detoxification superfamily |
|  | GRMZM2G306282 | | **-** | | | WAK5 | Wall associated kinase 5 [81-83] |
|  | GRMZM2G325477 | | **-** | | | PCR2 | Plant cadmium resistance 2 |
|  | GRMZM2G576752 | | **-** | | | WAK3 | Wall associated kinase 3, see cites for WAK5 |
| **Biotic stress** | GRMZM2G058872 | | **-** | | | ACD11 | Glycolipid transfer protein (GLTP) family |
|  | GRMZM2G163494 | | **-** | | | NRT3.1 | Nitrate transmembrane transporters |
|  | GRMZM2G173647 | | **-** | | | RPS2 | NB-ARC domain-containing disease resistance |
|  | GRMZM5G827121 | | **-** | | | RPS3 | NB-ARC domain-containing disease resistance protein |
| **RNA editing** | GRMZM2G129783 | | **-** | | | OTP84 | Tetratricopeptide repeat (TPR)-like superfamily protein |
| **Citric acid cycle/ETC** | GRMZM2G063909 | | **-** | | | CSY4 | Citrate synthase family protein |
|  | GRMZM2G171236 | | **-** | | | **-** | NADH-ubiquinone oxidoreductase-related, [84] |
|  | GRMZM2G109271 | | **-** | | | SDH1-1 | Succinate dehydrogenase 1-1 [85] |
|  | GRMZM2G306945 | | **-** | | | SDH1-1 | Succinate dehydrogenase 1-1 [85] |
