# Supplementary material for: Differentiated transcriptional signatures in the maize landraces of Chiapas, Mexico
Source: BMC Genomics. 2017 Sep 8;18:707. doi: 10.1186/s12864-017-4005-y (PMC5591509; doi:10.1186/s12864-017-4005-y)
Supplement: Supplementary file 5 — Raw, trimmed and mapped read counts for each of the 45 maize landrace RNA-seq libraries. (DOC 89 kb) [file 12864_2017_4005_MOESM5_ESM.doc]

Additional file 5: Raw, trimmed and mapped read counts for each of the 45 maize landrace RNA-seq libraries

| Elevation | ID | Raw counts | Trimmed paired-end | Trimmed singletons | Uniquely mapped reads  (0 mismatches) | | Uniquely mapped reads  (2 mismatches) |
| --- | --- | --- | --- | --- | --- | --- | --- |
| Lowland | 1.1 | 10,439,682 | 10,182,584 | 239,606 | 7,139,399 | 8,057,106 | |
|  | 1.2 | 10,430,532 | 9,782,834 | 569,155 | 6,740,973 | 7,794,724 | |
|  | 1.3 | 10,504,192 | 9,945,954 | 480,085 | 7,086,370 | 8,157,758 | |
|  | 4.1 | 11,292,632 | 11,011,414 | 261,899 | 7,831,552 | 8,874,598 | |
|  | 4.2 | 12,825,010 | 12,097,683 | 679,222 | 8,429,932 | 9,649,951 | |
|  | 4.3 | 10,330,470 | 9,765,568 | 486,097 | 7,115,540 | 8,142,660 | |
|  | 6.1 | 13,581,764 | 13,032,203 | 446,709 | 9,150,585 | 10,457,264 | |
|  | 6.2 | 20,737,732 | 19,587,880 | 1,038,222 | 11,991,434 | 13,825,068 | |
|  | 6.3 | 9,568,905 | 9,035,405 | 453,474 | 6,617,719 | 7,631,794 | |
|  | 7.1 | 11,153,956 | 10,881,011 | 252,581 | 7,986,770 | 9,019,609 | |
|  | 7.2 | 13,818,036 | 13,013,686 | 732,726 | 8,709,307 | 10,073,804 | |
|  | 7.3 | 9,462,968 | 8,884,115 | 474,976 | 6,410,195 | 7,411,220 | |
|  | 9.1 | 10,380,385 | 10,112,423 | 249,198 | 7,186,884 | 8,143,160 | |
|  | 9.2 | 13,513,444 | 12,755,328 | 691,518 | 9,311,219 | 10,726,555 | |
|  | 9.3 | 10,195,298 | 9,625,875 | 493,493 | 6,884,325 | 7,914,780 | |
| Midland | 10.1 | 10,588,864 | 10,250,995 | 304,751 | 7,495,939 | 8,521,976 | |
|  | 10.2 | 7,279,128 | 6,881,896 | 355,851 | 4,898,764 | 5,644,246 | |
|  | 10.3 | 9,845,739 | 9,276,558 | 499,067 | 6,334,315 | 7,348,480 | |
|  | 12.1 | 13,214,762 | 12,818,955 | 359,711 | 9,138,876 | 10,408,581 | |
|  | 12.2 | 9,991,418 | 9,674,662 | 277,799 | 6,815,411 | 7,763,058 | |
|  | 12.3 | 11,947,573 | 11,385,725 | 509,951 | 7,716,082 | 8,838,362 | |
|  | 13.1 | 13,083,726 | 12,648,770 | 394,578 | 9,064,717 | 10,346,368 | |
|  | 13.2 | 11,762,135 | 10,954,138 | 654,484 | 7,849,029 | 9,144,867 | |
|  | 13.3 | 11,967,217 | 11,346,672 | 573,631 | 7,729,959 | 8,962,273 | |
| Elevation | ID | Raw counts | Trimmed paired-end | Trimmed singletons | Uniquely mapped reads  (0 mismatches) | Uniquely mapped reads  (2 mismatches) | |
|  | 17.1 | 13,528,138 | 13,129,632 | 359,839 | 8,996,865 | 10,214,845 | |
|  | 17.2 | 11,949,060 | 11,568,709 | 332,091 | 8,218,826 | 9,367,518 | |
|  | 17.3 | 16,961,938 | 16,093,723 | 792,990 | 10,963,688 | 12,694,676 | |
|  | 18.1 | 13,683,294 | 13,236,512 | 387,945 | 9,607,943 | 10,948,525 | |
|  | 18.2 | 12,277,769 | 11,887,437 | 344,576 | 8,388,803 | 9,577,505 | |
|  | 18.3 | 5,292,851 | 5,015,296 | 250,365 | 3,581,160 | 4,125,075 | |
| Highland | 20.1 | 12,883,767 | 12,390,750 | 444,418 | 8,712,643 | 10,008,828 | |
|  | 20­­.2 | 12,242,627 | 11,886,034 | 321,072 | 8,251,586 | 9,432,015 | |
|  | 20­.3 | 14,205,156 | 13,461,901 | 637,821 | 9,566,940 | 11,007,865 | |
|  | 26.1 | 14,919,832 | 14,418,754 | 457,460 | 10,106,081 | 11,570,013 | |
|  | 26.2 | 12,723,433 | 12,339,342 | 340,536 | 8,289,458 | 9,455,402 | |
|  | 26.3 | 11,444,355 | 10,840,889 | 527,836 | 7,547,418 | 8,728,387 | |
|  | 27.1 | 13,297,370 | 12,839,493 | 417,909 | 9,189,327 | 10,546,302 | |
|  | 27.2 | 12,279,665 | 11,859,702 | 358,126 | 8,133,263 | 9,321,028 | |
|  | 27.3 | 12,208,517 | 11,622,856 | 537,065 | 8,028,978 | 9,294,031 | |
|  | 29.1 | 10,501,789 | 10,231,288 | 251,833 | 6,667,214 | 7,551,604 | |
|  | 29.2 | 13,590,231 | 13,130,261 | 407,067 | 9,584,572 | 10,961,216 | |
|  | 29.3 | 11,365,436 | 10,815,481 | 499,461 | 7,712,568 | 8,918,749 | |
|  | 30.1 | 14,952,693 | 14,396,962 | 501,474 | 10,230,359 | 11,774,007 | |
|  | 30.2 | 11,267,823 | 10,902,335 | 326,390 | 7,755,414 | 8,867,647 | |
|  | 30.3 | 12,536,177 | 11,944,892 | 544,556 | 7,699,196 | 8,864,515 | |

Read counts for each of the 45 libraries when first obtained from the sequencer (raw counts), after read preprocessing (trimmed paired end and trimmed singletons) and after mapping reads to the maize genome (uniquely mapped reads – zero mismatches and two mismatches). Paired end trimmed reads were scored separately from reads without a matching pair (singletons). Uniquely mapped reads were recorded when allowing both zero and two mismatches to determine if elevation of population origin influenced the number of reads that mapped to the B73 maize genome. The ID column includes three replicates for each of the 15 populations used in the experiment (i.e., 1.1, 1.2, 1.3 replicates 1-3 for population one).
