# Supplementary material for: Differentiated transcriptional signatures in the maize landraces of Chiapas, Mexico
Source: BMC Genomics. 2017 Sep 8;18:707. doi: 10.1186/s12864-017-4005-y (PMC5591509; doi:10.1186/s12864-017-4005-y)

Additional file 6: Thirty year precipitation (A) and evaporation (B) related environmental parameters of maize landrace origin. Landrace number and elevational zone are on the x-axis and environmental parameters are along the y-axis. Abbreviations: mm – millimeters, DM – daily mean, Max – maximum, M – mean, Min – minimum, MM – monthly mean, Normal_M – normal mean.


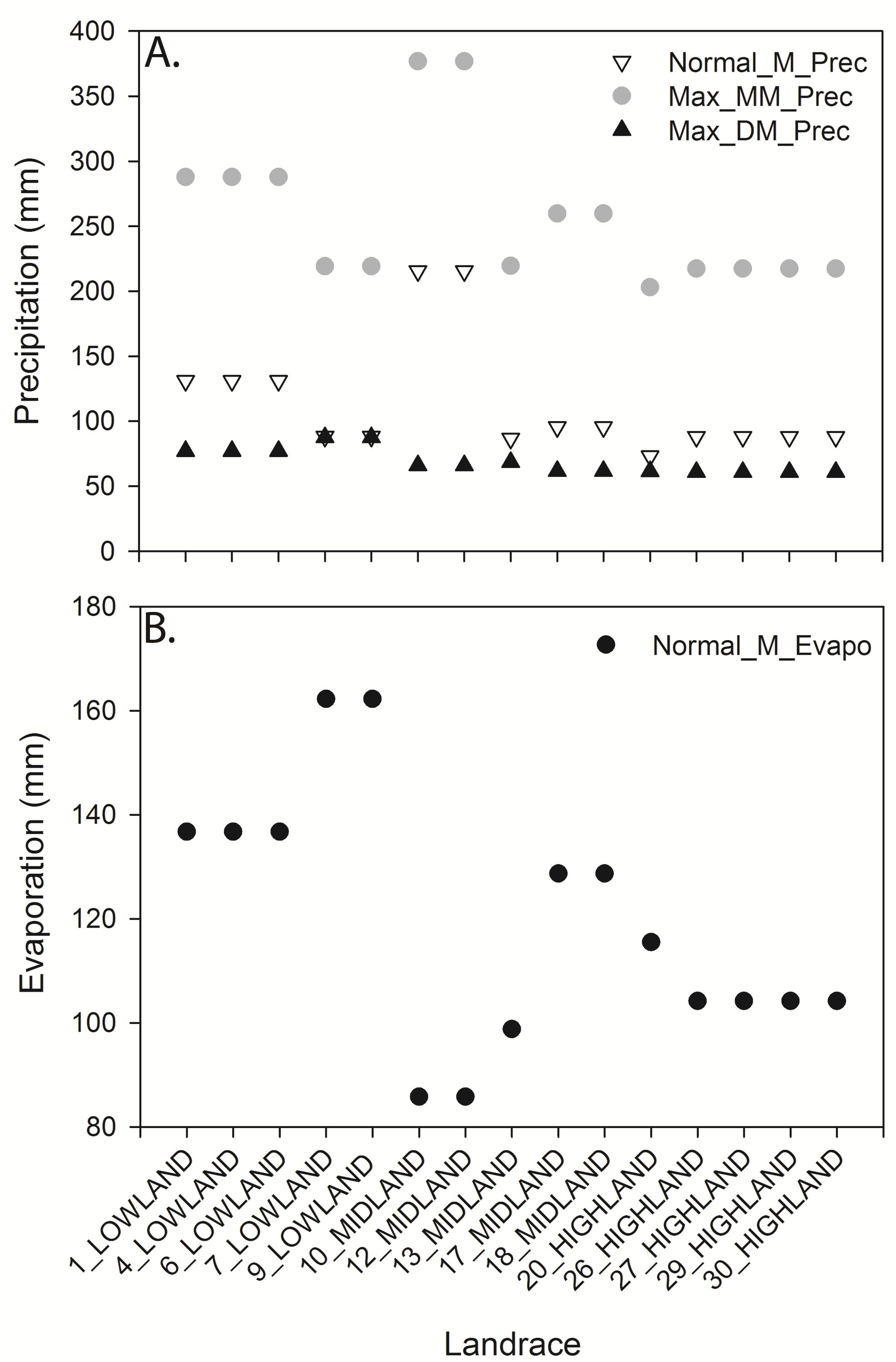

Supplement: Supplementary file 6 — Thirty year precipitation (A) and evaporation (B) related environmental parameters of maize landrace origin. (DOC 514 kb) [file 12864_2017_4005_MOESM6_ESM.doc]
