# Supplementary material for: Differentiated transcriptional signatures in the maize landraces of Chiapas, Mexico
Source: BMC Genomics. 2017 Sep 8;18:707. doi: 10.1186/s12864-017-4005-y (PMC5591509; doi:10.1186/s12864-017-4005-y)
Supplement: Supplementary file 7 — Environmental variation due to landscape variation. (DOC 22 kb) [file 12864_2017_4005_MOESM7_ESM.doc]

Additional file 7: Environmental variation due to landscape variation

The environmental data for lowland landraces 7 and 9 were obtained from the weather station in Aquespala, Frontera Comelapa, while that of the remaining three lowland landraces were collected from Chicomuselo, Chicomuselo (Table S1). Aquespala is located away from the eastern edge of the foothills of the Sierra Madre de Chiapas further into the Central Depression than Chicomuselo, which could have led to the decreased normal mean precipitation and increased evaporation in the region. The environments from which our midland landraces were collected appear to be most variable. Environmental data for midland landraces 10 and 12 were collected from a weather station in Tziscao, La Trinitaria (Table S1), which is surrounded by 59 lakes referred to as the Lagunas de Montebello (Montebello Lakes) that experiences relatively depressed temperatures, increased normal mean precipitation and decreased evaporation (Fig. 2). While the reasons behind the higher environmental parameter values for midland landraces 17 and 18 were less clear (Fig. 2), their difference from those for midland landrace 13 may have been due to the weather station for the former being located at a lower elevation.
