# Supplementary material for: Differentiated transcriptional signatures in the maize landraces of Chiapas, Mexico
Source: BMC Genomics. 2017 Sep 8;18:707. doi: 10.1186/s12864-017-4005-y (PMC5591509; doi:10.1186/s12864-017-4005-y)
Supplement: Supplementary file 8 — Co-expression module gene counts. (DOC 43 kb) [file 12864_2017_4005_MOESM8_ESM.doc]

Additional file 8: Co-expression module gene counts

| Ivory | 44 | Salmon | 716 |
| --- | --- | --- | --- |
| Lightcyan1 | 63 | Tan | 767 |
| Lightsteelblue | 71 | Greenyellow | 805 |
| Mediumpurple | 73 | Purple | 835 |
| Orangered | 75 | Magenta | 852 |
| Plum1 | 83 | Pink | 873 |
| Skyblue3 | 103 | Black | 900 |
| Yellowgreen | 120 | Red | 962 |
| Sienna3 | 127 | Green | 1030 |
| Darkmagenta | 128 | Yellow | 1862 |
| Darkolivegreen | 135 | Brown | 1889 |
| Violet | 140 | Blue | 4154 |
| Paleturquoise | 158 | Turquoise | 4470 |
| Stellblue | 165 | Grey | 4794 |
| Saddlebrown | 169 |  |  |
| Skyblue | 197 |  |  |
| White | 240 |  |  |
| Darkorange | 290 |  |  |
| Orange | 338 |  |  |
| Darkgrey | 478 |  |  |
| Darkgreen | 511 |  |  |
| Darkturquoise | 511 |  |  |
| Darkred | 535 |  |  |
| Royalblue | 546 |  |  |
| Lightyellow | 581 |  |  |
| Lightgreen | 613 |  |  |
| Grey60 | 670 |  |  |
| Lightcyan | 697 |  |  |
| Midnightblue | 702 |  |  |

Each of the 44 modules identified during module formation using WGCNA and the number of genes in each.
