# Supplementary material for: Differentiated transcriptional signatures in the maize landraces of Chiapas, Mexico
Source: BMC Genomics. 2017 Sep 8;18:707. doi: 10.1186/s12864-017-4005-y (PMC5591509; doi:10.1186/s12864-017-4005-y)
Supplement: Supplementary file 9 — GO enrichment analysis for the turquoise (A) and yellow (B) co-expression modules. (DOC 41 kb) [file 12864_2017_4005_MOESM9_ESM.doc]

Additional file 9: GO enrichment analysis for the turquoise (A) and yellow (B) co-expression modules

| **A. Turquoise module** | | | | |
| --- | --- | --- | --- | --- |
| **GO term** | **Ontology** | **Description** | **Input/Background** | **FDR** |
| GO:0034641 | BP | cellular nitrogen compound metabolic process† | 75/373 | 2.90E-06 |
| GO:0003824 | MF | catalytic activity† | 1120/11249 | 8.90E-05 |
| GO:0048475 | CC | coated membrane | 21/84 | 0.024 |
| GO:0030117 | CC | membrane coat† | 21/84 | 0.024 |
| **B. Yellow module** | | | | |
| **GO term** | **Ontology** | **Description** | **Input/Background** | **FDR** |
| GO:0000166 | MF | nucleotide binding | 198/3970 | 0.0074 |
| GO:0032559 | MF | adenyl ribonucleotide binding | 164/3197 | 0.0074 |
| GO:0032555 | MF | purine ribonucleotide binding | 180/3572 | 0.0074 |
| GO:0032553 | MF | ribonucleotide binding | 180/3572 | 0.0074 |
| GO:0005524 | MF | ATP binding† | 164/3193 | 0.0074 |
| GO:0004713 | MF | protein tyrosine kinase activity† | 78/1313 | 0.013 |
| GO:0017076 | MF | purine nucleotide binding | 184/3767 | 0.013 |
| GO:0005509 | MF | calcium ion binding† | 29/352 | 0.013 |
| GO:0001883 | MF | purine nucleoside binding | 168/3384 | 0.013 |
| GO:0001882 | MF | nucleoside binding | 168/3385 | 0.013 |
| GO:0016740 | MF | transferase activity | 177/3613 | 0.013 |
| GO:0004674 | MF | protein serine/threonine kinase activity† | 82/1430 | 0.013 |
| GO:0030554 | MF | adenyl nucleotide binding | 168/3384 | 0.013 |
| GO:0016616 | MF | oxidoreductase activity, acting on the CH-OH group of donors, NAD(P) as acceptor† | 23/241 | 0.013 |
| GO:0016614 | MF | oxidoreductase activity, acting on CH-OH group of donors | 23/270 | 0.032 |
|  |  |  |  |  |

Turquoise module (n=4470); yellow module (n=1862). Ontology column –ontology categories (BP, biological process; CC, cellular component; MF, molecular function); Description – description of overrepresented GO terms; Input/Background – # of genes in the input file/# of genes of that GO category in the reference gene set; FDR – false discovery rate for Fisher test using a Yekutieli multi-test adjustment to determine enriched GO category. † – descendent terms.
