# Supplementary material for: Differentiated transcriptional signatures in the maize landraces of Chiapas, Mexico
Source: BMC Genomics. 2017 Sep 8;18:707. doi: 10.1186/s12864-017-4005-y (PMC5591509; doi:10.1186/s12864-017-4005-y)
Supplement: Supplementary file 10 — Hub genes. (DOC 331 kb) [file 12864_2017_4005_MOESM10_ESM.doc]

Additional file 10: Hub genes: A) 85th percentile of module membership for the turquoise module and gene significance for the turquoise module – maximum normal mean temperature correlation; B) 85th percentile of module membership for the yellow module and gene significance for the yellow module – minimum daily mean temperature correlation. Maize gene IDs, Arabidopsis TAIR10 definition, and Best rice hit definition were obtained from Phytozome. The padj column contains Benjamini-Hochberg adjusted p-value (FDR) for each gene.

| **A. Turquoise module** | | |  |  |
| --- | --- | --- | --- | --- |
| **Maize gene ID** | **Arabidopsis TAIR10 definition** | **Best rice hit definition** | **log2FC** | **padj** |
| AC198725.4_FG007 | adaptin family protein | adaptin | 0.55, H | 1.00E-05 |
| AC204359.3_FG005 | Drug/metabolite transporter superfamily protein | DUF250 domain containing protein | 0.56, H | 0.0032 |
| GRMZM2G003225 | Nse4, Smc5/6 DNA repair complex | - | 1.82, H | 2.17E-07 |
| GRMZM2G005526 | Fe superoxide dismutase 3 | superoxide dismutase, chloroplast | 1.32, H | 0.00021 |
| GRMZM2G006964 | gibberellin 2-oxidase 8 | gibberellin 2-beta-dioxygenase 7 | -0.85, L | 5.15E-05 |
| GRMZM2G007140 | 2-oxoglutarate (2OG) and Fe(II)-dependent oxygenase superfamily protein | 1-aminocyclopropane-1-carboxylate oxidase homolog 4 | -0.42, L | 0.00024 |
| GRMZM2G008032 | - | - | 2.16, H | 2.36E-07 |
| GRMZM2G009785 | tocopherol cyclase, chloroplast / vitamin E deficient 1 (VTE1) / sucrose export defective 1 (SXD1) | tocopherol cyclase, chloroplast precursor | -0.36, L | 0.00024 |
| GRMZM2G010095 | - | - | 1.26, H | 1.74E-15 |
| GRMZM2G010282 | - | - | 2.29, H | 3.22E-09 |
| **GRMZM2G010920** | **myb-like HTH transcr. regulator family** | **Myb-like DNA-binding containing protein** | 1.26, H | 5.52E-14 |
| GRMZM2G013471 | - | - | 0.20, H | 0.16† |
| GRMZM2G017193 | Flavin-binding monooxygenase family protein | flavin monooxygenase | -1.13, L | 0.00032 |
| GRMZM2G018262 | Transmembrane amino acid transporter family protein | amino acid transporter | 1.62, H | 0.00081 |
| GRMZM2G019411 | pectin methylesterase 31 | pectinesterase | -0.92, L | 7.74E-08 |
| GRMZM2G020231 | - | - | -1.61, L | 0.0097 |
| GRMZM2G021471 | Fibronectin type III domain-containing protein | Fibronectin type III domain containing protein | 0.51, H | 2.65E-13 |
| GRMZM2G022506 | - | - | 1.00, H | 2.97E-11 |
| GRMZM2G022637 | Heavy metal transport/detoxification protein | heavy metal associated domain protein | -0.82, L | NA† |
| GRMZM2G024293 | P-loop containing nucleoside triphosphate hydrolases superfamily protein | ATP binding protein | -0.50, L | 8.96E-05 |
| GRMZM2G025173 | - | - | 3.12, H | 8.97E-17 |
| **A. Turquoise module cont.** | |  |  |  |
| **Maize gene ID** | **Arabidopsis TAIR10 definition** | **Best rice hit definition** | **log2FC** | **padj** |
| GRMZM2G027665 | C2 calcium/lipid-binding and GRAM domain | GRAM and C2 domains containing protein | -1.94, L | 5.74E-09 |
| **GRMZM2G033413** | **ABRE binding factor 4** | **bZIP transcription factor** | -2.36, L | 1.11E-26 |
| GRMZM2G034389 | peptide transporter 2 | peptide transporter PTR2 | -1.07, L | 0.00013 |
| **GRMZM2G035103** | **salt tolerance zinc finger** | **ZOS3-18 - C2H2 zinc finger protein** | -0.55, L | 0.018 |
| GRMZM2G035712 | - | - | -2.09, L | 0.00016 |
| GRMZM2G040605 | - | - | 1.59, H | 7.28-05 |
| GRMZM2G042981 | MBOAT (membrane bound O-acyl transferase) family | O-acyltransferase | 0.63, H | 7.11E-08 |
| GRMZM2G044143 | WD-40 repeat family protein / beige-related | beige/BEACH domain containing protein | 0.29, H | 0.0099 |
| GRMZM2G046933 | - | - | 1.06, H | 2.06E-07 |
| GRMZM2G045318 | Preprotein translocase Sec, Sec61-beta subunit | protein transport protein Sec61 subunit beta | 0.76, H | 1.76E-06 |
| GRMZM2G046924 | gamma carbonic anhydrase 1 | bacterial transferase hexapeptide domain protein | -0.31, L | 1.07E-08 |
| GRMZM2G049510 | Kinase interacting (KIP1-like) family protein | KIP1 | 0.70, H | 7.57E-05 |
| GRMZM2G053117 | MAP kinase 20 | CGMC_MAPKCMGC_2.9 - CGMC includes CDA, MAPK, GSK3, and CLKC kinases | 1.93, H | 7.67E-09 |
| GRMZM2G060344 | - | - | -2.51, L | 1.72E-05 |
| GRMZM2G061929 | - | - | -0.92, L | 1.92E-05 |
| GRMZM2G064371 | endoplasmic reticulum auxin binding protein 1 | auxin-binding protein 4 precursor | -0.94, L | 3.25E-13 |
| GRMZM2G064601 | - | - | -3.66, L | 7.92E-16 |
| **GRMZM2G067624** | **squamosa promoter binding protein-like 4** | **OsSPL13 - SBP-box gene family member** | -1.22, L | 0.00094 |
| GRMZM2G068808 | Terpenoid cyclases/Protein prenyltransferases superfamily | ent-kaurene synthase, chloroplast precursor | -1.05, L | 2.91E-22 |
| GRMZM2G069905 | - | - | -1.90, L | NA† |
| GRMZM2G070011 | Cysteine proteinases superfamily protein | vignain precursor | -0.73, L | 0.0012 |
| GRMZM2G071744 | - | - | 0.73, H | 0.23† |
| GRMZM2G072383 | - | - | -1.97, L | NA† |
| GRMZM2G073045 | S-adenosyl-L-methionine-dependent methyltransferases superfamily protein | methyltransferase | 0.32, H | 0.0034 |
| GRMZM2G074122 | phosphoenolpyruvate carboxylase 3 | phosphoenolpyruvate carboxylase | 0.58, H | 7.15E-08 |
| GRMZM2G074631 | UDP-glucosyl transferase 73B3 | UDP-glucoronosyl and UDP-glucosyl transferase | 2.10, H | 6.90E-21 |
| GRMZM2G076631 | alpha/beta-Hydrolases superfamily protein | hydrolase, alpha/beta fold family domain containing protein | -0.27, L | 0.0028 |
| GRMZM2G077206 | Glutathione S-transferase family protein | glutathione S-transferase GSTU6 | -0.57, L | 2.3E-07 |
| **A. Turquoise module cont.** | |  |  |  |
| **Maize gene ID** | **Arabidopsis TAIR10 definition** | **Best rice hit definition** | **log2FC** | **padj** |
| GRMZM2G077389 | alpha-galactosidase 2 | glycosyltransferase | -2.85, L | 3.06E-10 |
| GRMZM2G078508 | endoplasmic reticulum auxin binding protein 1 | auxin-binding protein 4 precursor | -0.67, L | 0.00031 |
| GRMZM2G079257 | Signal recognition particle, SRP54 subunit protein | signal recognition particle 54 kDa protein | -0.14, L | 0.055† |
| GRMZM2G079290 | - | phytosulfokines precursor | -1.68, L | 2.18E-07 |
| **GRMZM2G079727** | **AGAMOUS-like 8** | **OsMADS32 - MADS-box family gene with MIKCc type-box** | -2.49, L | 1.02E-05 |
| GRMZM2G080355 | stress enhanced protein 1 | - | -0.35, L | 0.00062 |
| GRMZM2G080439 | ubiquitin protein ligase 5 | HECT-domain domain containing protein | 0.17, H | 0.027 |
| GRMZM2G081585 | Fe superoxide dismutase 3 | superoxide dismutase, chloroplast | 1.32, H | 1.61E-06 |
| GRMZM2G082633 | alpha/beta-Hydrolases superfamily protein | lecithin cholesterol acyltransferase | -0.40, L | 0.014 |
| GRMZM2G088375 | ABC2 homolog 13 | ABC1 family domain containing protein | -1.53, L | NA† |
| GRMZM2G089574 | - | - | -1.45, L | 3.55E-07 |
| GRMZM2G093776 | poltergeist like 4 | protein phosphatase 2C | -0.66, L | 0.012 |
| GRMZM2G095323 | zinc finger (C2H2 type) family protein | ZOS12-03 - C2H2 zinc finger protein | 0.95, H | 9.72E-50 |
| GRMZM2G096153 | glutathione S-transferase F11 | glutathione S-transferase | 1.63, H | 2.62E-16 |
| **GRMZM2G097275** | **squamosa promoter binding protein-like 2** | **OsSPL3 - SBP-box gene family member** | 0.51, H | 3.11E-05 |
| GRMZM2G098319 | - | interacting protein of DMI3 | -1.91, L | 6.00E-06 |
| GRMZM2G100012 | cytochrome P450, family 709, subfamily B, polypeptide 2 | cytochrome P450 72A1 | -0.99, L | 0.38† |
| GRMZM2G101290 | Lactate/malate dehydrogenase family protein | lactate/malate dehydrogenase | 2.65, H | 7.66E-10 |
| GRMZM2G103617 | B12D protein | B12D protein | -0.91, L | 1.71E-10 |
| GRMZM2G108819 | - | - | -1.03, L | NA† |
| GRMZM2G109405 | purple acid phosphatase 29 | Ser/Thr protein phosphatase family protein | 1.46, H | 1.24E-10 |
| **GRMZM2G113078** | **Integrase-type DNA-binding superfamily protein** | **AP2 domain containing protein** | -1.21, L | 0.0058 |
| GRMZM2G115895 | - | - | -3.57, L | 6.89E-15 |
| GRMZM2G116584 | calcineurin B-like protein 10 | calcineurin B | -1.56, L | 3.92E-12 |
| GRMZM2G122239 | Cysteine proteinases superfamily protein | OTU-like cysteine protease family protein | 0.35, H | 5.39E-05 |
| GRMZM2G125455 | NHL domain-containing protein | NHL repeat-containing protein | -0.71, L | 0.00018 |
| GRMZM2G124175 | abscisic aldehyde oxidase 3 | aldehyde oxidase | -1.18, L | 8.45E-06 |
| GRMZM2G127635 | - | - | -1.12, L | 0.00053 |
| GRMZM2G133621 | CBS / octicosapeptide/Phox/Bemp1 (PB1) domains-containing protein | CBS domain-containing protein | 0.43, H | 7.11E-08 |
| GRMZM2G133793 | GNS1/SUR4 membrane protein family | GNS1/SUR4 membrane family protein | -0.95, L | 1.71E-10 |
| **A. Turquoise module cont.** | |  |  |  |
| **Maize gene ID** | **Arabidopsis TAIR10 definition** | **Best rice hit definition** | **log2FC** | **padj** |
| GRMZM2G135283 | serine transhydroxymethyltransferase 1 | serine hydroxymethyltransferase, mitochondrial precursor | 0.24, H | 0.0083 |
| GRMZM2G136306 | - | - | -0.29, L | 0.033 |
| GRMZM2G136455 | RAB GTPase homolog 1C | ras-related protein | 0.65, H | 0.0001 |
| **GRMZM2G138976** | **ARID/BRIGHT DNA-binding domain** | **AT-rich interaction region** | -0.54, L | 2.16E-14 |
| GRMZM2G141858 | Bifunctional inhibitor/lipid-transfer protein/seed storage 2S albumin superfamily protein | LTPL85 - Protease inhibitor/seed storage/LTP family protein precursor | -0.52, L | 0.0076 |
| GRMZM2G144028 | Protein kinase superfamily protein | OsWAK10d - OsWAK receptor-like cytoplasmic kinase OsWAK-RLCK | 0.65, H | 7.51E-06 |
| GRMZM2G144245 | pfkB-like carbohydrate kinase family protein | kinase, pfkB family | 1.35, H | 0.00011 |
| GRMZM2G147774 | cytochrome P450, family 72, subfamily A, polypeptide 15 | cytochrome P450 72A1 | -1.91, L | 0.0021 |
| GRMZM2G148167 | glucuronidase 3 | heparanase-like protein precursor | -0.82, L | NA† |
| GRMZM2G150656 | sterol carrier protein 2 | peroxisomal multifunctional enzyme type 2 | -0.58, L | 0.00011 |
| GRMZM2G154048 | - | - | -2.88, L | 1.40E-10 |
| **GRMZM2G154169** | **GRF1-interacting factor 3** | **GRF-interacting factor 2** | 0.49, H | 1.29E-06 |
| GRMZM2G159678 | Domain of unknown function (DUF303) | receptor protein kinase | -0.50, L | 0.0011 |
| GRMZM2G160853 | S-locus lectin protein kinase family protein | kinase | 1.53, H | 0.0041 |
| GRMZM2G167686 | Secretory carrier membrane protein (SCAMP) | - | -1.91, L | 3.09E-08 |
| GRMZM2G169412 | sorting nexin 1 | sorting nexin 1 | 0.97, H | 5.32E-12 |
| GRMZM2G172900 | NIMA-related kinase 5 | protein kinase domain containing protein | -0.45, L | 0.00018 |
| GRMZM2G174204 | Ribosomal protein S8e family protein | ribosomal protein | 1.73, H | NA† |
| GRMZM2G174560 | - | - | 2.58, H | NA† |
| GRMZM2G176301 | sterol 4-alpha-methyl-oxidase 2-2 | fatty acid hydroxylase | 2.31, H | 1.73E-14 |
| GRMZM2G177231 | Haloacid dehalogenase-like hydrolase (HAD) | - | -0.46, L | 2.39E-06 |
| GRMZM2G178693 | plasma membrane intrinsic protein 2 | aquaporin protein | -1.21, L | 0.0008 |
| GRMZM2G178756 | phosphoenolpyruvate carboxylase-relate kinase 1 | calcium-dependent protein kinase isoform AK1 | -0.30, L | 0.042 |
| GRMZM2G179551 | - | - | 0.65, H | 0.059† |
| GRMZM2G180988 | villin 2 | villin protein | 0.29, H | 0.0025 |
| GRMZM2G301885 | polyol/monosaccharide transporter 5 | transporter family protein | -0.55, L | 0.04 |
| GRMZM2G303995 | - | - | -1.47, L | 0.00047 |
| GRMZM2G304132 | - | - | -1.14, L | 0.0091 |
| GRMZM2G305757 | vacuolar H+-pumping ATPase 16 kDa proteolipid subunit 4 | - | -2.68, L | 1.86E-06 |
| **A. Turquoise module cont.** | |  |  |  |
| **Maize gene ID** | **Arabidopsis TAIR10 definition** | **Best rice hit definition** | **log2FC** | **padj** |
| GRMZM2G319760 | Histidine kinase-, DNA gyrase B-, and HSP90-like ATPase | ATP-binding region, ATPase-like domain containing protein | -1.08, L | 0.0011 |
| GRMZM2G320305 | ferredoxin-NADP(+)-oxidoreductase 2 | ferredoxin--NADP reductase, chloroplast precursor | -3.28, L | 4.8E-13 |
| GRMZM2G321023 | Rho GTPase activating protein with PAK-box/P21-Rho-binding domain | rhoGAP domain containing protein | 2.62, H | 7.40E-13 |
| GRMZM2G349565 | LRR and NB-ARC domains-containing disease resistance protein | rp1 | 1.19, H | 0.00013 |
| GRMZM2G351786 | UB-like protease 1D | ulp1 protease family protein | 1.35, H | 5.73E-08 |
| GRMZM2G360615 | - | fiber protein Fb34 | 1.63, H | 1.46E-16 |
| GRMZM2G369216 | endomembrane-type CA-ATPase 4 | calcium-transporting ATPase, endoplasmic reticulum-type | 1.74, H | 6.15E-10 |
| GRMZM2G370473 | ribosomal protein S19 | chloroplast 30S ribosomal protein S19 | 2.63, H | NA† |
| GRMZM2G373396 | - | - | 6.16, H | 3.90E-79 |
| GRMZM2G385186 | - | - | -3.28, L | 3.25E-10 |
| GRMZM2G386389 | - | - | 1.60, H | NA† |
| GRMZM2G393180 | - | - | -2.54, L | 3.77E-07 |
| **GRMZM2G398124** | **TATA binding protein associated factor 21kDa subunit** | **transcription initiation factor IID** | 2.20, H | 3.99E-09 |
| **GRMZM2G414141** | **transcription coactivators** | **TOPBP1C - Similar to DNA replication protein** | 1.52, H | 9.31E-07 |
| GRMZM2G419836 | Thioredoxin superfamily protein | - | 0.75, H | 6.26E-10 |
| GRMZM2G422210 | Cox19 family protein (CHCH motif) | - | 0.42, H | 0.00039 |
| GRMZM2G422537 | guanyl-nucleotide exchange factors; GTPase binding | - | -2.96, L | 6.41E-11 |
| GRMZM2G429842 | Subtilase family protein | OsSub57 - Putative Subtilisin homologue | -0.98, L | 0.0087 |
| GRMZM2G430526 | electron transfer flavoprotein alpha | electron transfer flavoprotein subunit alpha, mitochondrial precursor | -5.54, L | NA† |
| GRMZM2G435393 | Chalcone and stilbene synthase family protein | chalcone synthase | -1.56, L | 0.0073 |
| GRMZM2G436787 | general control non-repressible 3 | ABC transporter, ATP-binding protein | 2.45, H | 7.11E-08 |
| GRMZM2G439784 | disease resistance family protein / LRR family | leucine rich repeat protein | 1.57, H | 1.97E-11 |
| GRMZM2G440003 | methyl esterase 1 | OsPOP4 - Putative Prolyl Oligopeptidase homol. | 1.86, H | 2.47E-08 |
| GRMZM2G447806 | - | - | -1.32, L | 0.024 |
| GRMZM2G449779 | pumilio 23 | pumilio-family RNA binding repeat protein | 0.76, H | 1.52E-08 |
| GRMZM2G452930 | - | small G protein family protein | -1.74, L | NA† |
| GRMZM2G471814 | Phosphoribosyltransferase family protein | phosphoribosyl transferase | -3.51, L | 2.04E-13 |
| **A. Turquoise module cont.** | |  |  |  |
| **Maize gene ID** | **Arabidopsis TAIR10 definition** | **Best rice hit definition** | **log2FC** | **padj** |
| GRMZM2G473104 | pfkB-like carbohydrate kinase family protein | kinase, pfkB family | 1.13, H | 4.16E-05 |
| GRMZM2G474546 | Protein kinase superfamily protein | protein kinase domain containing protein | -0.47, L | 0.05 |
| GRMZM2G479000 | - | - | 1.38, H | 7.69E-05 |
| GRMZM2G485905 | - | - | -2.76, L | 9.96E-07 |
| GRMZM2G522533 | - | nucleolar GTPase | -2.70, L | 5.73E-07 |
| GRMZM2G539054 | - | - | 2.72, H | NA† |
| GRMZM2G565856 | galacturonosyltransferase 1 | glycosyl transferase family 8 | -2.65, L | NA† |
| GRMZM5G808811 | differentiation and greening-like 1 | DAG protein, chloroplast precursor | 0.31, H | 0.0019 |
| GRMZM5G816432 | uracil phosphoribosyltransferase | phosphoribosyl transferase | 1.39, H | 5.02E-47 |
| **GRMZM5G828396** | **basic helix-loop-helix (bHLH) DNA-binding superfamily protein** | **BHLH transcription factor** | 0.59, H | 0.0013 |
| GRMZM5G830269 | - | - | -2.10, L | 0.00031 |
| **GRMZM5G842484** | **high mobility group** | **SSRP1-like FACT complex subunit** | -1.58, L | 2.77E-05 |
| GRMZM5G843389 | RING/U-box superfamily protein | zinc finger, C3HC4 type domain protein | -0.48, L | 1.99E-05 |
| GRMZM5G852877 | plant uncoupling mitochondrial protein 1 | mitochondrial carrier protein | -0.65, L | 5.74E-07 |
| GRMZM5G862955 | cytochrome oxidase 2 | - | -1.24, L | NA† |
| GRMZM5G871520 | Protein kinase superfamily protein | serine/threonine-protein kinase | -0.79, L | 0.0037 |
| **GRMZM5G873335** | **ARID/BRIGHT DNA-binding domain** | **AT-rich interaction region** | -0.75, L | 5.34E-26 |
| GRMZM5G887286 | zinc ion binding;nucleic acid binding;zinc ion binding | ZOS11-03 - C2H2 zinc finger protein | -0.28, L | 0.0052 |
| GRMZM5G892308 | Thioredoxin superfamily protein | OsGrx_C12 - glutaredoxin subgroup III | -1.24, L | 0.0079 |
| GRMZM5G899825 | - | - | 1.08, H | NA† |

| **B. Yellow module** | | | | |
| --- | --- | --- | --- | --- |
| **Maize gene ID** | **Arabidopsis TAIR10 definition** | **Best rice hit definition** | **log2FC** | **Padj** |
| **AC186524.3_FG005** | **high mobility group B4** | **HMG1/2** | 3.33, H | 2.04E-13 |
| AC203101.3_FG002 | - | - | -0.98, L | 0.11† |
| AC206223.3_FG004 | transducin family / WD-40 repeat family | WD domain, G-beta repeat domain protein | 1.60, H | 0.00018 |
| GRMZM2G002542 | kinase family protein w/ leucine-rich repeat | receptor-like protein kinase 5 precursor | -1.48, L | 0.00019 |
| GRMZM2G003059 | calcium-dependent protein kinase 24 | CAMK_CAMK_like.46 - CAMK includes calcium/calmodulin dependent protein kinases\ | 1.47, H | NA† |
| GRMZM2G016487 | RING/U-box superfamily protein | zinc finger, C3HC4 type domain containing protein | 0.21, H | 0.0073 |
| GRMZM2G018558 | Cytosol aminopeptidase family protein | leucine aminopeptidase, chloroplast precursor | 2.46, H | 4.69E-07 |
| GRMZM2G018707 | - | - | -0.74, L | 0.0015 |
| GRMZM2G029101 | CSL zinc finger domain-containing protein | expp1 protein precursor | -0.59, L | 0.00049 |
| **B. Yellow module cont.** | |  |  |  |
| **Maize gene ID** | **Arabidopsis TAIR10 definition** | **Best rice hit definition** | **log2FC** | **Padj** |
| GRMZM2G030794 | - | - | -0.45, L | 0.022 |
| GRMZM2G033236 | - | - | -1.39, L | 0.00018 |
| GRMZM2G039886 | DNAJ heat shock family protein | dnaJ domain containing protein | 0.55, H | NA† |
| GRMZM2G043857 | Ankyrin repeat family protein | cadmium tolerance factor | -0.75, L | 0.003 |
| GRMZM2G044306 | RNA polymerase II large subunit | DNA-directed RNA polymerase III subunit RPC1 | 0.27, H | 0.00038 |
| GRMZM2G047616 | high-affinity K+ transporter 1 | OsHKT1;5 - Na+ transporter | -1.29, L | 7.15E-08 |
| **GRMZM2G052667** | **Integrase-type DNA-binding superfamily** | **AP2 domain containing protein** | **0.91, H** | **1.03E-06** |
| GRMZM2G053458 | ferredoxin 3 | 2Fe-2S iron-sulfur cluster binding domain containing protein | -0.34, L | 0.0023 |
| GRMZM2G053977 | NAD(P)-binding Rossmann-fold superfamily | short-chain dehydrogenase/reductase | -1.69, L | 1.55E-05 |
| GRMZM2G057983 | aluminum-activated malate transporter 9 | aluminum-activated malate transporter | 0.45, H | 0.00026 |
| GRMZM2G058872 | Glycolipid transfer protein (GLTP) family | GLTP domain containing protein | -0.46, L | 6.37E-07 |
| GRMZM2G059825 | Exostosin family protein | exostosin family domain containing protein | -0.40, L | 3.12E-05 |
| GRMZM2G060160 | tetratricopeptide repeat (TPR)-like superfamily | XPA-binding protein 2 | 0.26, H | 6.02E-05 |
| GRMZM2G060276 | F-box/RNI-like superfamily protein | OsFBL5 - F-box domain and LRR containing protein | 0.26, H | 0.034 |
| GRMZM2G063909 | citrate synthase family protein | citrate synthase | -2.04, L | 8.28E-28 |
| GRMZM2G065284 | arginine/serine-rich zinc knuckle-containing protein 33 | - | 3.53, H | 3.65E-12 |
| GRMZM2G065971 | magnesium transporter 4 | CorA-like magnesium transporter protein | -0.35, L | 0.00091 |
| GRMZM2G068947 | 12-oxophytodienoate reductase 1 | 12-oxophytodienoate reductase | 0.81, H | 6.37E-05 |
| GRMZM2G070575 | cycloartenol synthase 1 | cycloartenol synthase | -0.77, L | NA† |
| **GRMZM2G077197** | **regulatory protein (NPR1)** | **BTBA1 - Bric-a-Brac,Tramtrack, Broad Complex BTB domain with Ankyrin repeat region** | **-0.71, L** | **7.39E-05** |
| GRMZM2G080487 | - | keratin, type I cytoskeletal 9 | -1.13, L | 1.51E-09 |
| GRMZM2G087556 | - | - | -1.48, L | 0.003 |
| GRMZM2G088737 | - | transmembrane protein | 0.59, H | 6.90E-12 |
| GRMZM2G092451 | DNA topoisomerase 1 beta | DNA topoisomerase 1 | 0.20, H | 3.69E-06 |
| **GRMZM2G098227** | **ARM repeat protein interacting with ABF2** | **ABTB1 - Armadillo repeats with a Bric-a-Brac, Tramtrack, Broad Complex BTB domain** | 0.19, H | 0.0022 |
| GRMZM2G108527 | - | - | 4.15, H | NA† |
| GRMZM2G109271 | succinate dehydrogenase 1-1 | succinate dehydrogenase flavoprotein subunit,mitochondrial precursor | -1.75, L | 2.65E-08 |
| **B. Yellow module cont.** | |  |  |  |
| **Maize gene ID** | **Arabidopsis TAIR10 definition** | **Best rice hit definition** | **log2FC** | **Padj** |
| GRMZM2G111920 | - | - | -1.43, L | 0.002 |
| GRMZM2G117642 | - | - | 0.04, H | 0.9† |
| GRMZM2G118243 | pleiotropic drug resistance 4 | pleiotropic drug resistance protein | -0.79, L | 0.0031 |
| GRMZM2G119714 | protein kinase superfamily protein | Protein kinase domain containing protein | -0.78, L | 8.25E-06 |
| GRMZM2G121024 | Heavy metal transport/detoxification superfamily | heavy metal-associated domain containing protein | -1.48, L | 2.40E-13 |
| GRMZM2G123107 | beta-1,3-glucanase 1 | glycosyl hydrolases family 17 | -0.75, L | 0.079† |
| GRMZM2G125268 | aldehyde dehydrogenase 2B4 | aldehyde dehydrogenase | -0.92, L | 0.0017 |
| GRMZM2G126858 | Leucine-rich repeat transmembrane protein kinase | SHR5-receptor-like kinase | 0.55, H | NA† |
| GRMZM2G129783 | tetratricopeptide repeat (TPR)-like superfamily protein | pentatricopeptide | -0.36, L | 0.011 |
| GRMZM2G137435 | phosphoinositide-specific phospholipase C | phospholipase C | -0.33, L | 0.024 |
| GRMZM2G139157 | protein kinase superfamily protein | tyrosine protein kinase domain containing protein | 3.24, H | NA† |
| GRMZM2G157505 | - | EGG APPARATUS-1 | 1.86, H | 5.10E-11 |
| GRMZM2G158248 | GRAM domain family protein | - | 0.41, H | 0.00018 |
| GRMZM2G163494 | nitrate transmembrane transporters | high affinity nitrate transporter | -0.93, L | 0.00063 |
| GRMZM2G166099 | - | - | 1.37, H | NA† |
| GRMZM2G168807 | - | major ampullate spidroin 2-2 | 0.25, H | 0.048 |
| GRMZM2G171028 | - | Plant viral response family protein | -1.59, L | 0.00047 |
| GRMZM2G171236 | NADH-ubiquinone oxidoreductase-related | mitochondrial NADH-ubiquinone oxidoreductase | -0.83, L | 1.66E-05 |
| GRMZM2G173647 | NB-ARC domain-containing disease resistance | disease resistance protein RPS2 | -0.60, L | 0.00036 |
| GRMZM2G178640 | - | - | 2.45, H | 8.60E-07 |
| GRMZM2G178641 | - | - | 2.82, H | 5.12E-09 |
| GRMZM2G181607 | atypical CYS HIS rich thioredoxin 4 | thioredoxin | -1.43, L | 0.0023 |
| GRMZM2G306282 | wall associated kinase 5 | OsWAK33 - OsWAK receptor-like protein OsWAK-RLP | -2.41, L | 3.33E-12 |
| GRMZM2G306945 | succinate dehydrogenase 1-1 | succinate dehydrogenase flavoprotein subunit,mitochondrial precursor | -3.28, L | 1.86E-20 |
| **GRMZM2G308034** | **myb domain protein 103** | **MYB family transcription factor** | 1.81, H | 0.0037 |
| GRMZM2G310144 | ABC2 homolog 4 | ABC transporter, ATP-binding protein | -1.93, L | 1.54E-07 |
| GRMZM2G316474 | Leucine-rich repeat protein kinase family | leucine-rich repeat family protein | -0.50, L | 3.24E-05 |
| GRMZM2G316907 | Leucine-rich repeat protein kinase family | receptor-like protein kinase 2 precursor | 1.34, H | 0.00021 |
| **B. Yellow module cont.** | |  |  |  |
| **Maize gene ID** | **Arabidopsis TAIR10 definition** | **Best rice hit definition** | **log2FC** | **Padj** |
| GRMZM2G323422 | - | - | -0.97, L | 3.78E-06 |
| GRMZM2G325477 | plant cadmium resistance 2 | uncharacterized Cys-rich domain protein | -1.61, L | 1.53E-07 |
| **GRMZM2G327349** | **WRKY family transcription factor** | **WRKY9** | -0.99, L | 0.00011 |
| GRMZM2G343769 | inhibitor/interactor with cyclin-dependent kinase | cyclin-dependent kinase inhibitor | 1.62, H | 0.0025 |
| GRMZM2G377641 | triglyceride lipases;triglyceride lipases | lipase class 3 family protein | 0.22, H | 0.026 |
| GRMZM2G403162 | - | - | 2.08, H | 4.31E-26 |
| GRMZM2G403719 | cysteine-rich RLK (RECEPTOR-like protein kinase) 23 | TKL_IRAK_DUF26-lc.26 - DUF26 kinases have homology to DUF26 containing loci | 1.69, H | 3.53E-09 |
| GRMZM2G415359 | lactate/malate dehydrogenase family protein | lactate/malate dehydrogenase | 0.28, H | 0.00027 |
| GRMZM2G416498 | Tetratricopeptide repeat (TPR)-like | Rf1, mitochondrial precursor | 0.48, H | 0.0087 |
| GRMZM2G418515 | modifier of snc1 | - | 0.22, H | 0.0013 |
| GRMZM2G420694 | - | NADH-ubiquinone oxidoreduct. 51 kDa subunit, mitochondrial precursor | 1.59, H | NA† |
| GRMZM2G428391 | heat shock protein 70 | DnaK family protein | 0.59, H | NA† |
| GRMZM2G459610 | - | - | -0.89, L | 0.00055 |
| GRMZM2G459702 | Pseudouridine synthase family protein | pseudouridylate synthase | 0.37, H | 0.00067 |
| GRMZM2G475948 | receptor lectin kinase | receptor like protein kinase | -0.97, L | 5.76E-05 |
| GRMZM2G487318 | SIT4 phosphatase-associated family protein | guanylyl cyclase | 1.88, H | NA† |
| GRMZM2G493136 | - | - | 1.35, H | NA† |
| GRMZM2G518717 | calcineurin-like metallo-phosphoesterase | Ser/Thr protein phosphatase family protein | 0.34, H | 5.21E-05 |
| GRMZM2G576752 | wall associated kinase 3 | OsWAK63 - OsWAK receptor-like protein kinase | -1.33, L | 4.89E-10 |
| GRMZM2G704005 | Lactoylglutathione lyase / glyoxalase I family protein | glyoxalase family protein | -0.34, L | 0.018 |
| GRMZM5G813606 | - | - | 2.29, H | 2.89E-14 |
| GRMZM5G826321 | alpha/beta-Hydrolases superfamily protein | alpha/beta hydrolase fold | -0.80, L | 9.86E-06 |
| GRMZM5G827121 | NB-ARC domain-containing disease resistance protein | resistance protein | -1.31, L | 0.00019 |
| GRMZM5G858887 | Cyclic nucleotide-regulated ion channel family protein | cyclic nucleotide-gated ion channel 2 | 0.28, H | 0.002 |

Hyphens, nonexistent data/uncharacterized; log2FC, log2 fold change for highland to lowland comparison (H, up-regulated in the highlands landraces; L, up-regulated in the lowland landraces); padj, adjusted p value; †, not differentially expressed at a 0.05 FDR. Bold gene names represent transcription factors.
