# Supplementary material for: Differentiated transcriptional signatures in the maize landraces of Chiapas, Mexico
Source: BMC Genomics. 2017 Sep 8;18:707. doi: 10.1186/s12864-017-4005-y (PMC5591509; doi:10.1186/s12864-017-4005-y)
Supplement: Supplementary file 11 — Genes making up the turquoise module overrepresented GO categories coated membrane and membrane coat. (DOC 42 kb) [file 12864_2017_4005_MOESM11_ESM.doc]

Additional file 11: Genes making up the turquoise module overrepresented GO categories coated membrane and membrane coat

| **Maize gene ID** | **L2FC (H/L)** | **L2FC (M/L)** | **Arabidopsis TAIR10 definition** | **Best rice hit definition** |
| --- | --- | --- | --- | --- |
| AC198725.4_FG007 | 0.547432982* | 0.5237* | adaptin family protein | adaptin |
| GRMZM2G010054 | 0.093195737 | 0.1435* | Coatomer, alpha subunit | coatomer alpha subunit, putative, expressed |
| GRMZM2G012501 | 0.304383389* | 0.4589* | coatomer gamma-2 subunit, putative / gamma-2 coat protein | coatomer subunit gamma-1, putative, expressed |
| GRMZM2G017421 | 0.236842463* | 0.2128* | Adaptor protein complex AP-1, gamma subunit | AP-1 complex subunit gamma-1, putative, expressed |
| GRMZM2G029077 | 0.18647777* | 0.1543 | adaptin family protein | adaptin, putative, expressed |
| GRMZM2G030144 | 0.202980751* | 0.2923* | Adaptor protein complex AP-1, gamma subunit | AP-1 complex subunit gamma-1, putative, expressed |
| GRMZM2G031360 | 0.035699523 | 0.1170 | Clathrin adaptor complexes medium subunit family protein | adaptor complexes medium subunit family domain containing protein, expressed |
| GRMZM2G045987 | 0.116979366 | 0.2042* | Sec23/Sec24 protein transport family protein | protein transport protein, putative, expressed |
| GRMZM2G049641 | 0.046057739 | 0.1596 | SNARE-like superfamily protein | clathrin adaptor complex small chain domain containing protein, expressed |
| GRMZM2G052713 | 0.213371066 | 0.2663 | SNARE-like superfamily protein | clathrin adaptor complex small chain domain containing protein, expressed |
| GRMZM2G054210 | 0.359079457* | 0.3392* | Adaptor protein complex AP-1, gamma subunit | AP-1 complex subunit gamma-1, putative, expressed |
| GRMZM2G054378 | 0.750115534* | 0.3940 | tRNA (guanine-N-7) methyltransferase | tRNA methyltransferase, putative, expressed |
| GRMZM2G057576 | 0.252710709 | 0.3214* | Clathrin, heavy chain | clathrin heavy chain, putative, expressed |
| GRMZM2G063775 | -0.172806626 | -0.1066 | Sec23/Sec24 protein transport family protein | protein transport protein, putative, expressed |
| GRMZM2G079201 | -0.833980198 | -0.2666 | Clathrin adaptor complexes medium subunit family protein | adaptor complexes medium subunit family domain containing protein, expressed |
| GRMZM2G095124 | 0.131480802* | 0.1503* | Adaptin family protein | adapitin protein, putative, expressed |
| GRMZM2G141587 | 0.086693633 | 0.1268 | Coatomer, beta\' subunit | coatomer subunit beta, putative, expressed |
| GRMZM2G143725 | 0.187495253 | 0.2235* | Sec23/Sec24 protein transport family protein | protein transport protein Sec24-like, putative, expressed |
| GRMZM2G146697 | 0.106528973 | 0.1078 | Adaptin family protein | adaptin, putative, expressed |
| GRMZM2G149406 | 0.099748325 | 0.1446 | Coatomer, alpha subunit | coatomer alpha subunit, putative, expressed |
| GRMZM2G378906 | 0.164874368* | 0.2299* | Coatomer, beta subunit | coatomer subunit beta-1, putative, expressed |

Positive numbers for L2FC (H/L) were up-regulated in the highlands; positive numbers for L2FC (M/L) were up-regulated in the midlands. Those with * were significantly differentially expressed.
